# Regulation and role of the ER stress transcription factor CHOP in alveolar epithelial type-II cells

**DOI:** 10.1007/s00109-019-01787-9

**Published:** 2019-04-26

**Authors:** Oleksiy Klymenko, Martin Huehn, Jochen Wilhelm, Roxana Wasnick, Irina Shalashova, Clemens Ruppert, Ingrid Henneke, Stefanie Hezel, Katharina Guenther, Poornima Mahavadi, Christos Samakovlis, Werner Seeger, Andreas Guenther, Martina Korfei

**Affiliations:** 1 Department of Internal Medicine, Justus-Liebig-University Giessen, Klinikstrasse 36, 35392 Giessen, Germany; 2 German Center for Lung Research (DZL), Universities of Giessen and Marburg Lung Center (UGMLC), 35392 Giessen, Germany; 3 Excellence Cluster Cardiopulmonary System (ECCPS), 35392 Giessen, Germany; 4 Department of Molecular Biosciences, The Wenner-Gren Institute, Stockholm University, SE-106 91 Stockholm, Sweden; 5 Department of Lung Development and Remodeling, Max-Planck-Institute for Heart and Lung Research, 61231 Bad Nauheim, Germany; 6 European IPF Network and European IPF Registry, Giessen, Germany; 7 Agaplesion Lung Clinic Waldhof-Elgershausen, 35753 Greifenstein, Germany

**Keywords:** Endoplasmic reticulum (ER) stress, C/EBP homologous protein (CHOP), Type-II alveolar epithelial cells (AECII), Transcriptional regulation, Activator protein-1 (AP-1), Protein c-Ets-1

## Abstract

**Abstract:**

Idiopathic pulmonary fibrosis (IPF) is a fatal disease characterized by type-II alveolar epithelial cell (AECII) injury and fibroblast hyperproliferation. Severe AECII endoplasmic reticulum (ER) stress is thought to underlie IPF, but is yet incompletely understood. We studied the regulation of C/EBP homologous protein (CHOP), a proapoptotic ER-stress-related transcription factor (TF) in AECII-like cells. Interestingly, single or combined overexpression of the active ER stress transducers activating transcription factor-4 (Atf4) and activating transcription factor-6 (p50Atf6) or spliced x-box-binding protein-1 (sXbp1) in MLE12 cells did not result in a substantial *Chop* induction, as compared to the ER stress inducer thapsigargin. Employing reporter gene assays of distinct *CHOP* promoter fragments, we could identify that, next to the conventional amino acid (AARE) and ER stress response elements (ERSE) within the *CHOP* promoter, activator protein-1 (AP-1) and c-Ets-1 TF binding sites are necessary for *CHOP* induction. Serial deletion and mutation analyses revealed that both AP-1 and c-Ets-1 motifs act in concert to induce *CHOP* expression. In agreement, *CHOP* promoter activity was greatly enhanced upon combined versus single overexpression of AP-1 and c-Ets-1. Moreover, combined overexpression of AP-1 and c-Ets-1 in MLE12 cells alone in the absence of any other ER stress inducer was sufficient to induce Chop protein expression. Further, AP-1 and c-Ets-1 were upregulated in AECII under ER stress conditions and in human IPF. Finally, Chop overexpression in vitro resulted in AECII apoptosis, lung fibroblast proliferation, and collagen-I production. We propose that *CHOP* activation by AP-1 and c-Ets-1 plays a key role in AECII maladaptive ER stress responses and consecutive fibrosis, offering new therapeutic prospects in IPF.

**Key messages:**

- Overexpression of active ER stress sensors Atf4, Atf6, and Xbp1 does not induce *Chop*.
- AP-1 and c-Ets-1 TFs are necessary for induction of the ER stress factor *Chop*.
- AP-1 and c-Ets-1 alone induce Chop expression in the absence of any ER stress inducers.
- AP-1 and c-Ets-1 are induced in AECII under ER stress conditions and in human IPF.
- Chop expression alone triggers AECII apoptosis and consecutive profibrotic responses.

**Electronic supplementary material:**

The online version of this article (10.1007/s00109-019-01787-9) contains supplementary material, which is available to authorized users.

## Introduction

Idiopathic pulmonary fibrosis (IPF) is an irreversible progressive lung disease, with a median diagnosis at 66 years and an estimated survival of 3–4 years following diagnosis [1, 2]. Excessive alveolar epithelial cell (AEC) death, abnormal fibroblast proliferation, and cumulative deposition of extracellular matrix (ECM) lead to alveolar destruction in the IPF lungs. With the exception of lung transplantation, there is no curative treatment available for IPF [1–3]. Thus, a better understanding of IPF pathogenesis is urgently needed to develop new treatment modalities for this fatal disease.

A growing body of evidence implicates injury and apoptosis of type-II AEC (AECII) as critical events in IPF pathogenesis [4, 5] which lead to activation and accumulation of fibroblasts and their transdifferentiation into contractile, ECM-producing myofibroblasts [6, 7]. However, the molecular mechanisms leading to fibrogenesis in response to AECII death still remain elusive. In a variety of preceding studies, “maladaptive” proapoptotic endoplasmic reticulum (ER) stress has been reported in the AECII of patients with sporadic and familial IPF [8–10]. In familial cases, this may be caused by mutations in the surfactant protein (SP)-C (*SFTPC*) and SP-A (*SFTPA*) genes, which cause misfolding of SP-C and SP-A proteins, respectively [9, 11–13]. In sporadic cases, the reasons are yet unclear, but various conditions such as oxidative stress, DNA damage, aging, protein overload, viral infections, and expression of damaged/mutant proteins may well contribute to ER stress and unfolded protein response (UPR) induction [14, 15]. If ER homeostasis is not restored, the UPR may also induce a maladaptive ER stress response through a variety of pathways, of which upregulation of the proapoptotic transcription factor C/EBP homologous protein (CHOP) has been suggested to represent an important one [16–18]. Upon induction and nuclear translocation, CHOP upregulates the transcription of proapoptotic factors such as BH-3 only (*BIM*) [17] and death receptor 5 (*DR5*) [15, 18] and downregulates anti-apoptotic genes such as B-cell leukemia/lymphoma 2 protein (*BCL2*) [19]. In addition, CHOP has been implicated in exaggerated reactive oxygen species (ROS) production by upregulation of the UPR-regulated oxidative protein folding machinery (through overexpression of ER oxidoreductin-1α, ERO1α) in the ER, which is directly contributing to ROS generation through the oxidation of disulfide bonds [20].

Induction and nuclear overexpression of CHOP in AECII is an eminent feature in IPF lungs and is detected together with other ER stress markers, such as activating transcription factor-4 (ATF4) [PRKR-like endoplasmic reticulum kinase (PERK) pathway], p50ATF6 [activating transcription factor-6 (ATF6) pathway], and spliced x-box-binding protein-1 (XBP1) [inositol-requiring protein-1α (IRE1α) pathway], as well as caspase-3 activation [8].

The transcriptional induction of *CHOP* during ER stress is regulated through four *cis*-acting elements in the *CHOP* promoter, namely by two amino acid response elements AARE1 and AARE2, and by two ER stress response elements ERSE1 and ERSE2 [14, 15, 21, 22]. The AARE sites can be activated by the PERK/ATF4 branch of the UPR [21–23], whereas the ERSE sites can be activated through both the IRE1α/XBP1 and/or the ATF6 pathway [21, 24, 25]. Such view, however, results in the conundrum, how the very same signaling pathways (the three UPR branches consisting of the IRE1α, PERK, and ATF6 pathways) should cause either adaptive ER stress resulting in cellular survival or maladaptive ER stress resulting in apoptosis of the cell. We, therefore, hypothesized that additional regulatory elements may be of importance in the transcriptional regulation of *CHOP* expression upon severe ER stress. We also speculated that alveolar epithelial induction of the proapoptotic ER stress factor CHOP alone is capable of triggering AECII apoptosis with consecutive promotion of fibroproliferative responses.

## Materials and methods

### Cell culture experiments

AECII-like MLE12 (mouse) and A549 (human) cells, kidney-epithelial HEK293T cells, and murine fibroblast cell line Mlg were all obtained from ATCC. The isolation of primary murine AECII from the lungs of C57BL/6J mice including their culture is outlined in detail in Online Resource 1. The animal experimental procedures for the isolation of primary AECII from lungs of C57BL/6J were approved by the institutional animal welfare representative and the local governmental ethics committee for animal welfare (Regierungspräsidium Giessen, Germany).All cell culture experiments, including the generation of stably transfected epithelial cell lines MLE12/pBI-L-CHOP and MLE12/pBI-L-EV (empty vector control), are provided in Online Resource 1.

### Bioinformatic analysis of the *CHOP* promoter

The genomic sequence of the 5′ upstream flanking region of the human *CHOP* gene was retrieved from Ensemble (Accession No. ENSG00000175197). Sequence analysis seeking for putative transcription factor binding sites was performed using MatInspektor (Genomatix) and TFSEARCH software (www.cbrc.jp/research/db/TFSEARCH).

### Cloning, vector constructs, and mutagenesis

For cloning of murine genes, cDNA was generated by reverse transcription from RNA extracts of normal murine lung tissue from C57Bl/6 mice. Murine full-length cDNAs for *Atf6* (p50Atf6), *Atf4*, *Chop*, *Mzf1*, *Sp1*, *Jun*, and *Ets1* were then amplified by PCR using gene-specific primers (Online Resource 1, Table S1) and HotStar-HighFidelity Polymerase (Qiagen). The template for amplification of spliced *Xbp1* was the plasmid *TETO***/***CMV***/***pUC19***-***Xbp1* (obtained from Prof. T. Weaver, Cincinnati, OH). *Chop* cDNA was cloned into the bidirectional pBI-L-vector (Clontech) or the adenoviral vector pAdTrack-CMV (Addgene). The cDNAs for *Mzf1*, *Sp1*, *Jun*, and *Ets1* were cloned into the pCMV-3Tag-4-myc expression vector (Agilent Technologies). The cDNAs for *Atf6*, spliced *Xbp1*, and *Atf4* were cloned into the expression vector pIRES2-DsRed2 (Clontech). The DNA fragments of the human *CHOP* and *ACTB* promoter were amplified from genomic DNA of normal human lung tissue; gene-specific primers for amplification are shown in Online Resource 1, Table S2. For serial deletion analysis, small genomic DNA sequences from the 4th fragment of the human *CHOP* promoter were amplified using the whole DNA sequence of the 4th fragment as template; the respective cloning primers are listed in Online Resource 1, Table S3. All promoter fragments as well as small genomic DNA sequences were amplified using the HotStarTaq DNA Polymerase (Qiagen), followed by cloning into the pGL4.14 [luc2/hygro]-Luciferase Reporter vector (Promega). Mutagenesis of AP-1 and c-Ets-1 DNA binding sites on the human *CHOP* promoter was performed using the QuickChange Site-Directed Mutagenesis Kit (Stratagene) and specific mutagenesis primers which are listed in Online Resource 1, Table S4. All constructs were verified by DNA sequencing (GATC Biotech). In general, A549, MLE12, or HEK293T cells were transfected with expression vector constructs/*CHOP*-*Luciferase* promoter constructs using TurboFect reagent (Thermo Scientific) according to the manufacturer’s instructions. Cells were harvested after the indicated time points to either isolate protein or RNA for further analyses, or for measuring *Luciferase* activity.

### Luciferase assay

Cells in 12-well plates were transfected with 900 ng of various generated *CHOP*-*Luciferase* promoter constructs and 100 ng of *β-Galactosidase* vector. After 24 h, cells were treated with vehicle (0.02% dimethyl sulfoxide, DMSO) or 2 μg/ml tunicamycin for 2 h. Thereafter, *Luciferase* assay and *β-Galactosidase* assay (both Promega) were performed according to the manufacturer’s instructions and are outlined in detail in Online Resource 1.

### Standard methodology

Full details for immunoblotting, immunohistochemistry (IHC), immunofluorescence (IF), quantitative (q)RT-PCR, chromatin-immunoprecipitation (ChIP), cell culture experiments including generation of stably transfected *Chop*-MLE12 cells, and infection of primary AECII with adenoviral vectors are available in Online Resource 1. The primers used for qRT-PCR analyses are listed in Table S5 of Online Resource 1.

### Microarray analysis

Transcriptome analysis of MLE12 cells in response to thapsigargin (TG) treatment and to single or combined overexpression of Atf4, p50Atf6, or (s)Xbp1 was performed using 4 × 44K 60mer oligonucleotide spotted microarray slides (Mouse Whole Genome 4 × 44K, Agilent Technologies, P/N G4122F). Full details are available in Online Resource 1. Microarray data have been uploaded to GEO on September 23, 2016. The accession number is GSE87298. To access the data, use the following link: https://www.ncbi.nlm.nih.gov/geo/query/acc.cgi?token=efqzsaymdzkvbun&acc=GSE87298.

### Human lung tissue

Lung tissue samples were obtained from seven patients with sporadic IPF (mean age ± SD 49.83 ± 7.60 years; three females, four males) and five nondiseased control subjects (organ donors; mean age ± SD 47.20 ± 11.03 years; one female, four males). Explanted lungs or lobes were obtained from the Department of Thoracic Surgery, Vienna, Austria (W. Klepetko) and were collected in the frame of the European IPF Registry/Biobank (eurIPFreg/bank). Biomaterials were provided by the UGMLC Giessen Biobank, member of the DZL Platform Biobanking. The study protocol was approved by the Ethics Committee of the Justus-Liebig-University Giessen (Nos. 111/08 and 58/15), and informed consent was obtained in written form from each subject. All IPF diagnoses were made on the basis of the IPF consensus guidelines [2].

### Statistics

Data were analyzed by GraphPad Prism 5.02 software and are expressed as mean ± SD. Statistical significance of differences between two groups was evaluated by unpaired Student’s *t* test. For the statistical comparison of differences between *n* ≥ 3 groups, one-way ANOVA with Bonferroni’s multiple comparison test as posttest was applied. Data were checked for normal distribution by Shapiro–Wilk test with the use of Origin7G. Significance level is indicated by *P < 0.05, **P < 0.01, and ***P < 0.001.

## Results

### Overexpression of ER stress transducers Atf6, Atf4, and sXbp1 is not sufficient to upregulate *Chop*

**Fig. 1:**
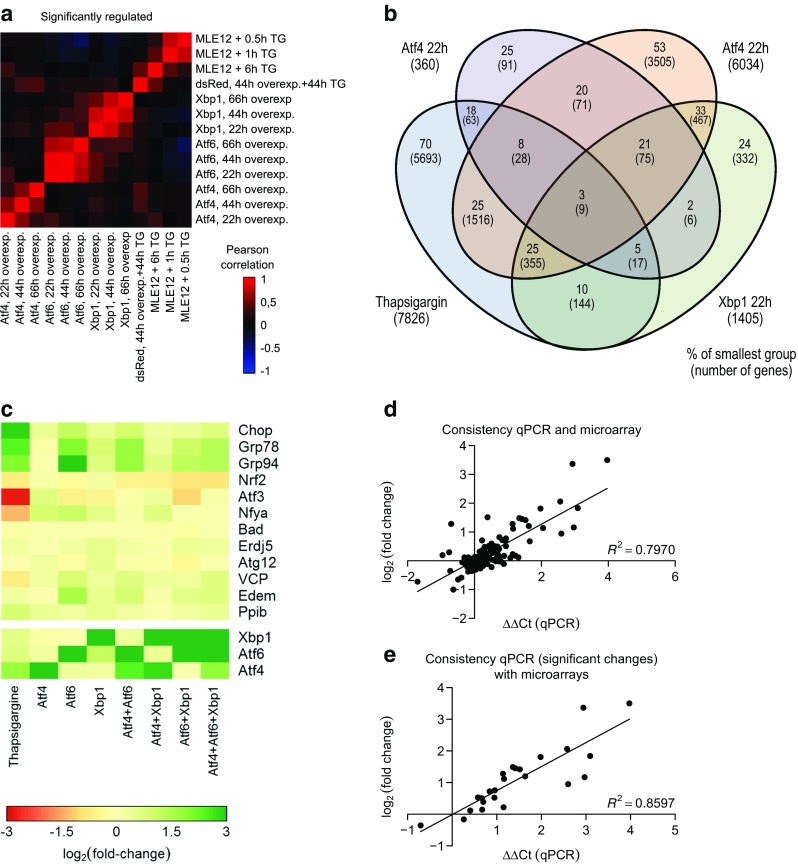
Cellular effects elicited by overexpression of Atf4, p50Atf6 and sXbp1 or by exposure to thapsigargin (TG). **a** Whole genome expression analysis using RNA from MLE12 cells overexpressing Atf4, p50Atf6, or sXbp1, versus empty vector (dsRed) and thapsigargin (TG, 1 μM/ml)-treated cells (*n* = 6 experiments per condition for time periods 22, 44, and 66 h; and *n* = 4 for the 0.5-, 1-, and 6-h treatments with TG; and *n* = 12 for the respective control). Only significantly regulated genes are included (based on a 5% false-discovery rate). The color coding (red = Pearson correlation coefficient of 1; blue = Pearson correlation coefficient of − 1) indicates the extent of correlation between the different conditions. **b** Venn diagram depicting the numbers of genes regulated significantly 22 h after transfection and their relative distribution in the different subgroups (same *n* numbers as in **a**). **c** Heatmap for gene regulation of ER stress and apoptosis markers in MLE12 cells in response to single or combined overexpression of Atf4, p50Atf6, and sXbp1 and thapsigargin treatment, against the empty vector at 22 h after transfection. From real-time PCR data, dCt values were calculated as dCt = Ct[reference] − Ct[target gene] using the mean Ct of *Actb*, *B2m*, and *Hmbs* as reference genes. All Ct values were measured in triplicate, the mean dCt values were determined from two independent experiments. The log_2_ fold changes are given by the ddCt values, where ddCt = mean dCt[treatment] − mean dCt[empty vector transfection]. Green and red indicate increased and decreased gene expression, respectively. **d**, **e** The changes in gene expression by qPCR of the genes are plotted against the changes as assessed by microarrays. A linear regression line and the corresponding indicator R show the linear dependency of both values

According to the current literature, *CHOP* expression can be induced by the PERK/ATF4, ATF6, and IRE1α/XBP1 pathways in response to severe ER stress [15, 21–25]. However, in our hands, single or combined transfection of a murine lung epithelial cell line (MLE12) mimicking AECII characteristics with plasmids expressing the active ER stress transducers Atf4, p50Atf6, or spliced (s)Xbp1 in vitro caused distinct and partially overlapping transcriptional changes, and it did not result in a substantial *Chop* induction (Fig. 1a–e and Online Resource 2, Fig. S1). In contrast, treatment of MLE12 cells with TG, a well-known maladaptive ER stress inducer, caused substantial *Chop* induction (Fig. 1a–e and Online Resource 2, Fig. S1); 73% of the differentially regulated genes under TG treatment were not encountered to be differently regulated upon transfection with Atf4, p50Atf6, or sXbp1 (Fig. 1a, b). The gene set enrichment analysis revealed that overexpression of either Atf4, p50Atf6, or sXbp1 or treatment with TG affected different and only partially overlapping sets of pathways, indicating a broad spectrum of cellular responses to ER stress (Online Resource 1, Table S6). We validated the microarray analyses by qPCR for several ER stress genes including *Chop* (Fig. 1c–e); and again, the effect of *Chop* induction in response to overexpression of ER stress transducers was minimal as compared to the induction by TG (Fig. 1c). Conversely, RNAi-based gene silencing of *Atf4* and *Atf6*, either alone or together, in MLE12 cells did not cause a significant reduction in the *Chop* level in response to TG treatment, despite dampened *Atf4* and *Atf6* expression (Online Resource 2, Fig. S2a–c). The Ire1α-mediated *Xbp1* splicing was not impaired in response to *Atf4* and *Atf6* knockdown (Online Resource 2, Fig. S2d). Taken together, these results suggested the presence of uncharacterized regulators of *Chop* in addition to the conventional UPR in AECII/AECII-like tumor cell lines.

### *CHOP* promoter can be activated in the absence of ERSE and AARE elements

**Fig. 2:**
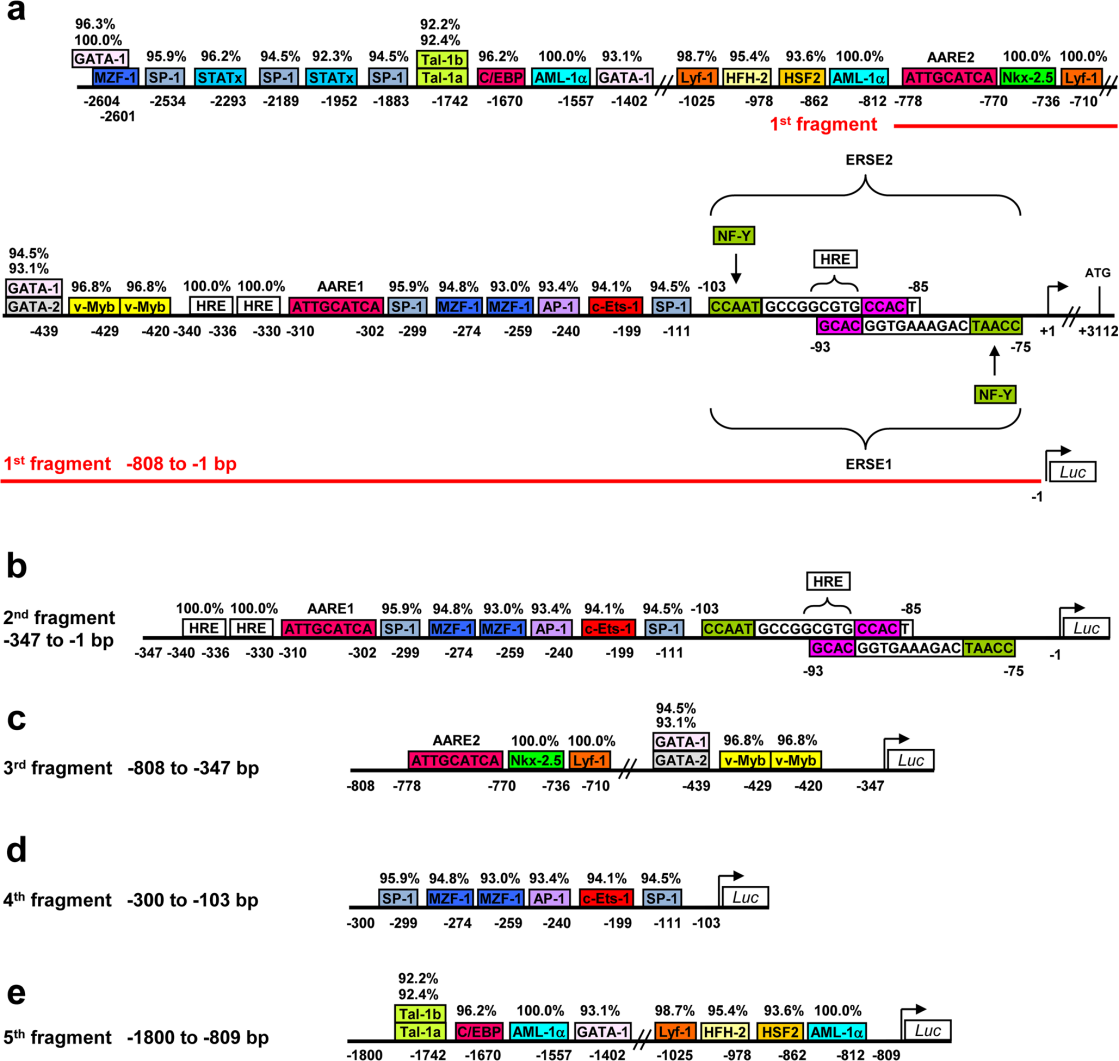
In silico analysis of the human *CHOP* promoter. **a** Schematic illustration of the 2.7-kb 5′ flanking region of the human *CHOP* gene. TFSEARCH and MatInspektor software were used to identify putative transcription factor (TF) binding sites in silico. The transcription initiation site (bent arrow) and translation site (ATG) are shown. Only high scoring (> 90%) transcription factor binding sites in the 2.7-kb 5′-flanking region of the human *CHOP* gene are depicted, in addition to the known and well-conserved ERSE (ER stress response element) and AARE (amino-acid response element) TF binding sites. **b** Schematic structure of the five amplified *CHOP* promoter fragments analyzed in this study, cloned into the pGL4.14-*Luciferase* Reporter vector. Abbreviations: NF-Y = nuclear transcription factor Y; SP-1 = transcription factor SP-1; c-Ets-1 = protein c-Ets-1 (or p54); AP-1 = activator protein 1 (or c-Jun); MZF-1 = myeloid zinc finger 1; HRE = hypoxia response element; v-Myb = transcriptional activator v-Myb; GATA = GATA family of transcription factors (GATA1 and GATA2); Lyf-1 = lymphoid transcription factor Lyf-1 or DNA-binding protein Ikaros; Nkx-2.5 = homeobox protein Nkx-2.5; AML-1α = acute myeloid leukemia 1 protein or Runt-related transcription factor 1; HSF2 = heat shock factor 2; HFH2 = hepatocyte nuclear factor 3 forkhead homolog 2; C/EBP = CCAAT/enhancer-binding protein; Tal-1a/1b = T-cell acute lymphocytic leukemia protein 1a/1b; STATx = signal transducer and activator of transcription

To identify such potential regulators, we performed an in silico analysis of the 2.7-kb 5′-flanking region of the human *CHOP* gene. We found 34 putative *cis*-regulatory transcription factor (TF) binding sites for 17 different transcription factors including the two well-known AARE sites (AARE2: bases − 778 to 770; AARE1: bases − 310 to − 302) and the two ERSE elements (bases − 103 to − 76) in reversed orientations [14, 15, 22–25] (Fig. 2a and Online Resource 2, Fig. S3). To investigate the transcriptional regulation of the human *CHOP* gene, five different fragments of the *CHOP* promoter were amplified by PCR. Figure 2a–e shows the schematics of the five amplified promoter fragments with their respective regulatory elements. We were particularly interested in the 4th and 5th fragment (Fig. 2d, e), as these did not include any ERSE and AARE elements. Further, we performed reporter gene assays in the presence of the ER-stress-inducing agent tunicamycin (TM), because it revealed *Atf4*/*Atf6* responses similar to TG, but more robust *Chop* induction as compared to TG (Online Resource 2, Fig. S4a).

**Fig. 3:**
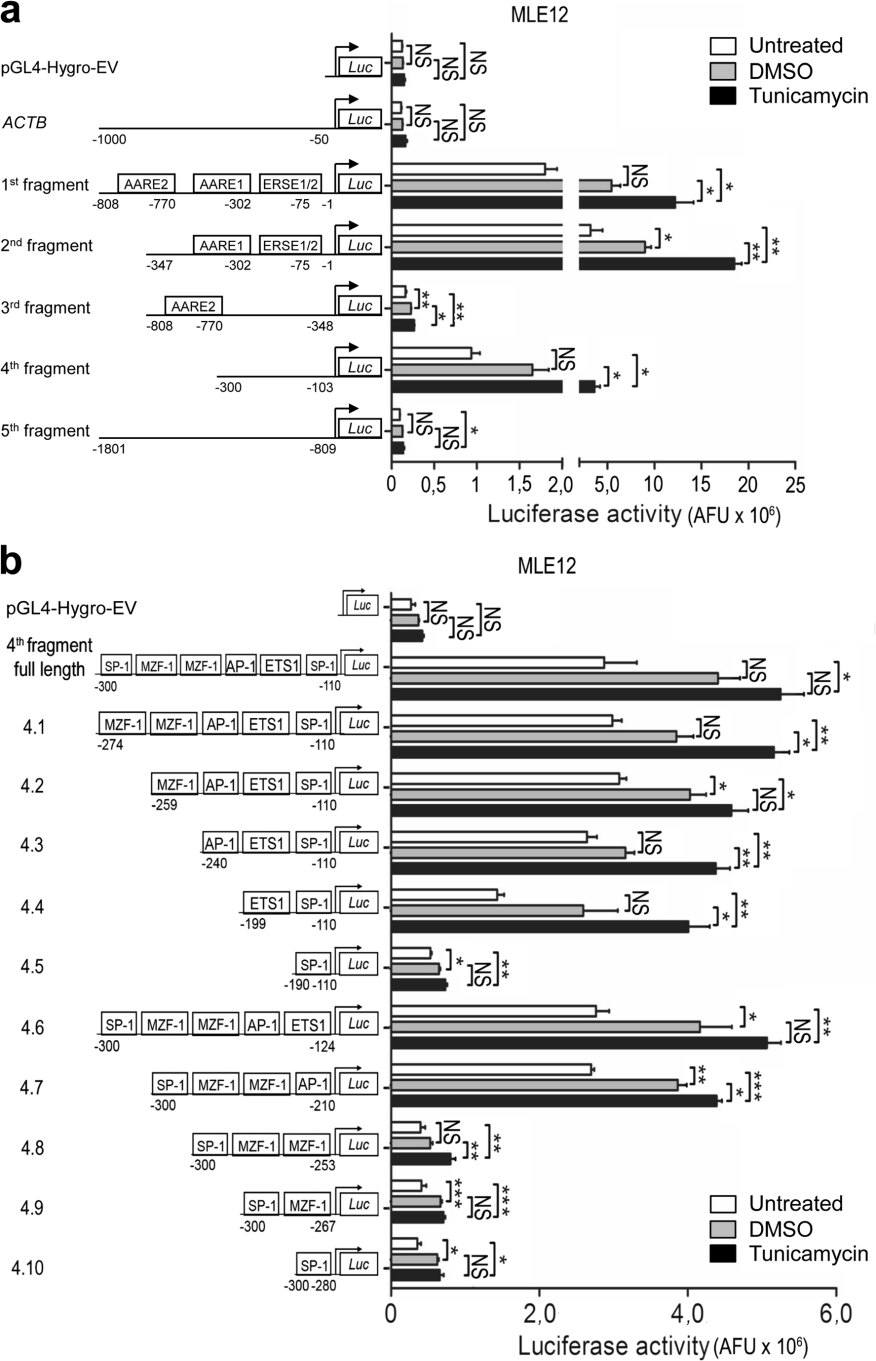
Analysis of distinct *CHOP* promoter constructs by *Luciferase* reporter gene assays. **a***Luciferase* reporter gene assays of cloned promoter constructs containing five different fragments of the human *CHOP* promoter or one *ACTB* promoter fragment. All fragments including pGL4-Hygro empty vector (EV) were transiently transfected together with a vector containing a *β-Galactosidase* reporter gene into MLE12 cells, followed by tunicamycin (2 μg/ml) or vehicle (0.02% DMSO) treatment 24 h after transfection. After 2 h, *Luciferase* and *β-Galactosidase* assays were performed as described in the “Materials and methods” section. Results are presented as normalized *Luciferase* activity. Means ± SD are shown, from *n* = 3 independent experiments, with analysis by Bonferroni’s multiple comparison test. *P < 0.05, **P < 0.01, NS = nonsignificant. **b** Deletion analysis of the 4th fragment of the human *CHOP* promoter: In each construct, one of the TF binding sites was deleted from 5′ to 3′ direction (constructs 4.1–4.5) and from 3′ to 5′ direction (constructs 4.6–4.10) in the 4th fragment of the human *CHOP* promoter. All small genomic DNA fragments were generated by PCR amplification using the whole DNA sequence of the 4th fragment as template. Cloned fragments including pGL4-Hygro empty vector (EV) were then transiently transfected together with a vector containing a *β-Galactosidase* reporter gene into MLE12 cells, followed by tunicamycin (2 μg/ml) or vehicle (0.02% DMSO) treatment 24 h after transfection. After 2 h, *Luciferase* and *β-Galactosidase* assays were performed as described in the “Materials and methods” section. Results are presented as normalized *Luciferase* activity. Means ± SD are shown, from *n* = 3 independent experiments, with analysis by Bonferroni’s multiple comparison test. *P < 0.05, **P < 0.01, ***P < 0.001, NS = nonsignificant

Reporter gene assays in lung epithelial A549 and MLE12 as well as in kidney HEK293T cells, which all showed Chop induction in response to TM treatment (Online Resource 2, Fig. S5a–f), revealed a high transcriptional activity for the 1st and 2nd promoter fragment (containing both ERSE and AARE elements) in nonstressed cells. This activity was greatly enhanced upon stimulation with TM, as compared to an irrelevant *ACTB* promoter fragment and empty vector transfection serving as negative controls (Fig. 3a and Online Resource 2, Fig. S5g and Fig. S5h). In contrast, the 3rd (containing AARE2) and the 5th fragments (with no ERSE or AARE elements) displayed a weak promoter activity comparable to the negative controls. Importantly, the 4th promoter fragment, lacking the ERSE and AARE elements, showed a significant increase in promoter activity in all three epithelial cell lines (Fig. 3a and Online Resource 2, Fig. S5g and Fig. S5h). These results suggested that some of the TF binding sites of the 4th fragment of the human *CHOP* promoter may significantly contribute to the transcriptional regulation of the *CHOP* gene upon ER stress.

### AP-1 and c-Ets-1 concomitantly regulate *CHOP* expression in lung epithelial cells

**Fig. 4:**
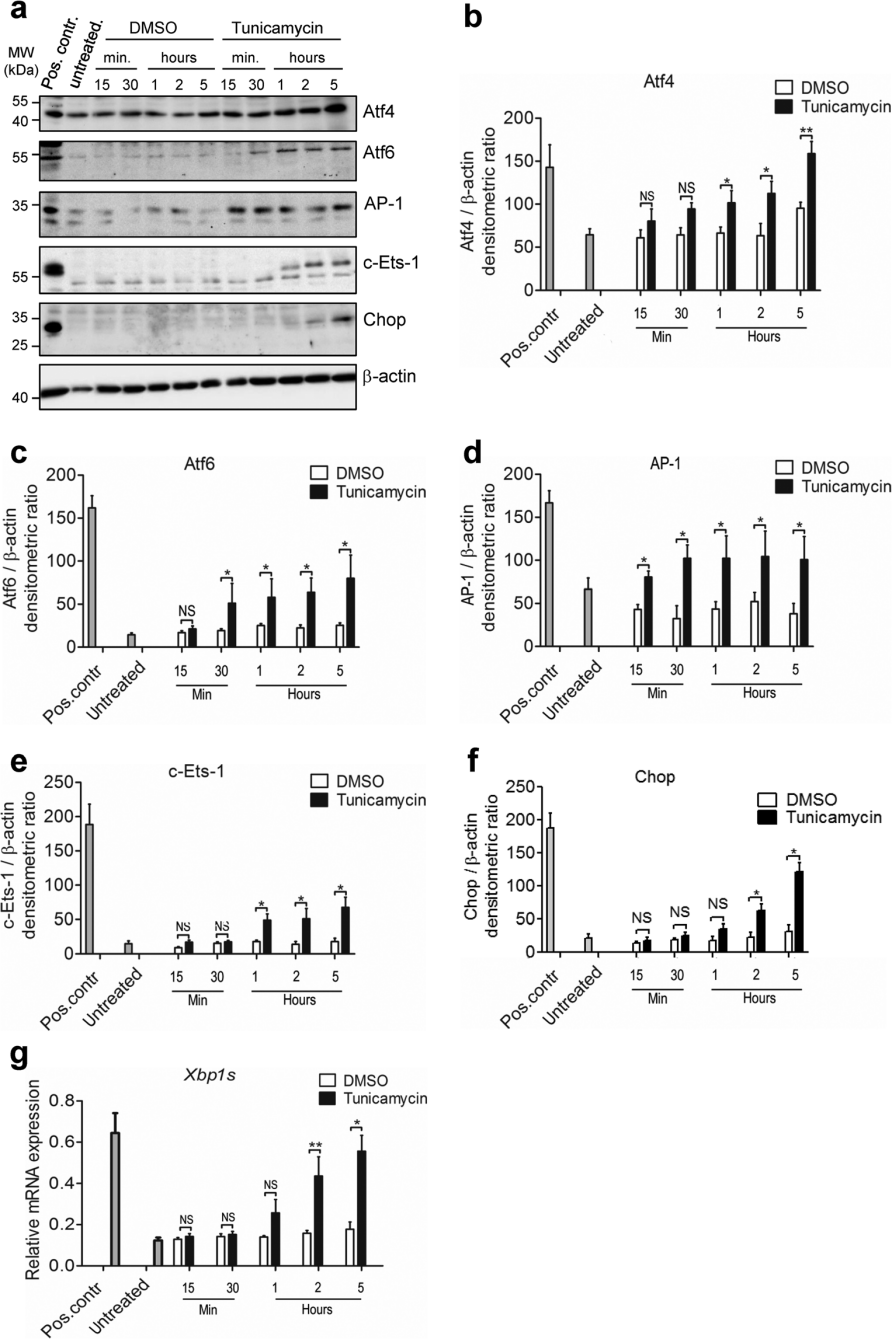
AP-1 and c-Ets-1 are upregulated under ER stress conditions. **a**–**f** MLE12 cells were treated with 0.02% DMSO (vehicle) or 2 μg/ml tunicamycin for the indicated time periods. Protein lysates were subjected to western blotting for indicated antibodies. **a** The protein levels for Atf4 (**b**), Atf6 (**c**), AP-1 (**d**), c-Ets-1 (**e**), and Chop (**f**) were densitometrically quantified by using ImageJ software. For normalization, β-actin expression was used as a control. All data are expressed as means ± SD, from *n* = 3 independent experiments, with analysis by unpaired Student’s *t* test. *P < 0.05, **P < 0.01, NS = nonsignificant. **g** Expression of spliced *Xbp1* mRNA in MLE12 cells in response to 2 μg/ml tunicamycin. The *Actb* gene was used as reference gene. Data are expressed as means ± SD, from *n* = 3 independent experiments, with analysis by unpaired Student’s *t* test. *P < 0.05, **P < 0.01, NS = nonsignificant. Data information: Pos. contr. = positive control, 24 h treatment of MLE12 cells with 1 μg/ml of tunicamycin

To identify the transcriptionally relevant TF binding sites present in the 4th fragment, we performed a serial deletion analysis of the 4th fragment of the human *CHOP* promoter. In each construct, one of the TF binding sites was deleted from 5′ to 3′ (constructs 4.1–4.5 in Fig. 3b) or from 3′ to 5′ direction (constructs 4.6–4.10 in Fig. 3b). *Luciferase* activity of the different deletion constructs indicated the importance of AP-1 (bases − 246 to − 240) and ETS1/c-Ets-1 (bases − 205 to − 199) binding sites (which lie in juxtaposition with each other on the 4th fragment) in regulating *Luciferase* activity. In contrast, the SP-1 and MZF-1 binding sites did not affect promoter activity (see constructs 4.3 and 4.6–4.10 in Fig. 3b). Based on these results, we hypothesized that AP-1 and c-Ets-1 TFs physically interact with each other to bind to the 4th fragment of the human *CHOP* promoter under severe ER stress conditions. Following the treatment of MLE12 cells with TM, AP-1 and c-Ets-1 were significantly upregulated together with the ER stress markers Atf4, Atf6, Chop, and spliced *Xbp1* (Fig. 4a–g), confirming that maladaptive ER stress resulted in overexpression of AP-1 and c-Ets-1. A similar ER stress response was observed in primary mouse AECII in response to TM treatment (Online Resource 2, Fig. S6). It should also be noted that, according to our transcriptome analysis, *Jun* (AP-1) and *Ets1* (c-Ets-1) were not induced in response to single Atf4, Atf6, or sXbp1 overexpression as compared to TG-treated cells (Online Resource 2, Fig. S7).

**Fig. 5:**
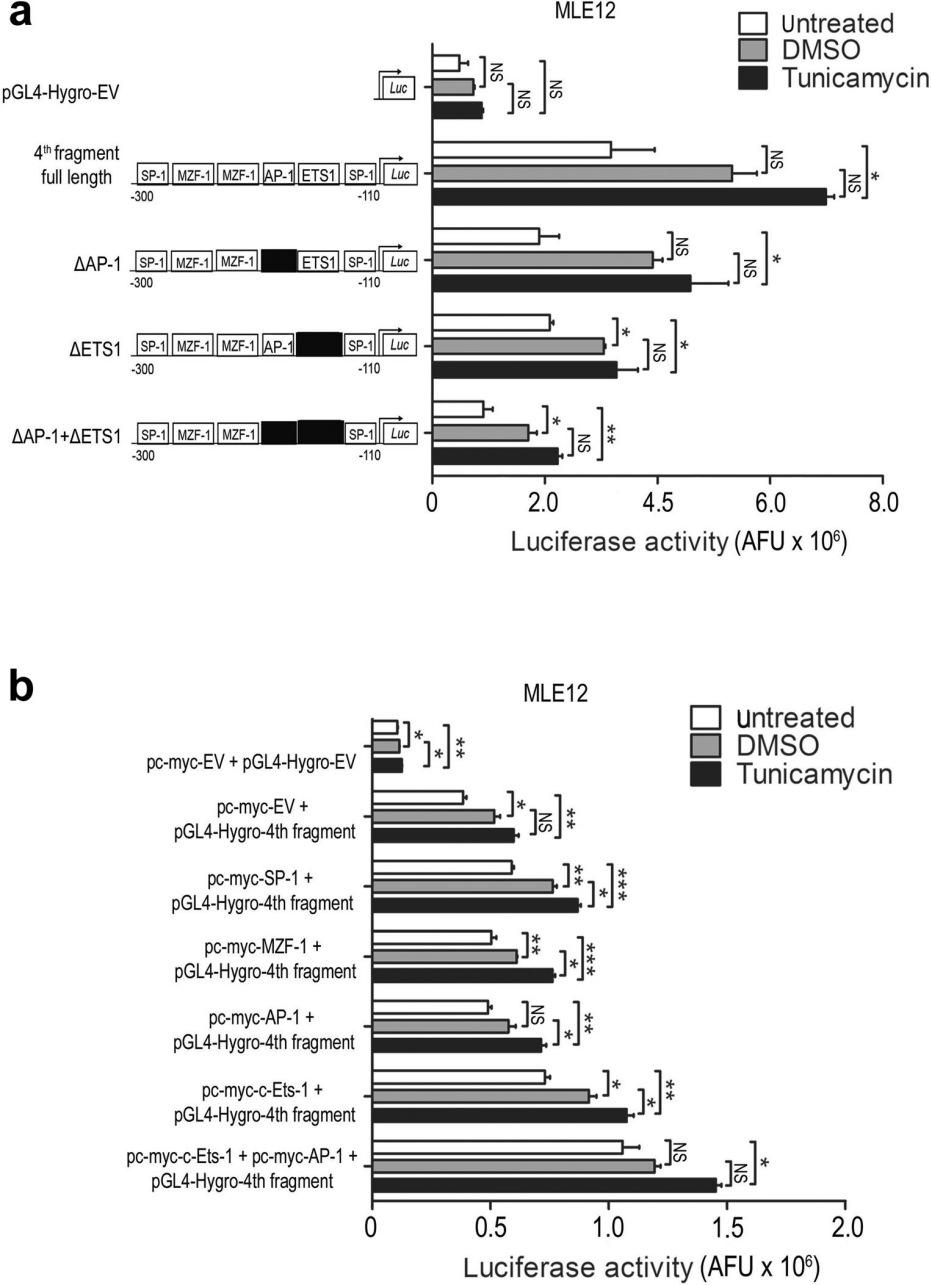
Requirement of AP-1 and c-Ets-1 binding sites for activation of the human *CHOP* promoter. **a** Impaired reporter gene activity in response to mutated AP-1 (ΔAP-1) and/or c-Ets-1 (ΔETS1) transcription factor binding sites in the 4th fragment of the *CHOP* promoter. All constructs including pGL4-Hygro empty vector (EV) were transiently transfected together with a vector containing a *β-Galactosidase* reporter gene into MLE12 cells, followed by tunicamycin (2 μg/ml) or vehicle (0.02% DMSO) treatment 24 h after transfection. After 2 h, *Luciferase* and *β-Galactosidase* assays were performed as described in the “Materials and methods” section. Results are presented as normalized *Luciferase* activity. Means ± SD are shown, from *n* = 3 independent experiments, with analysis by Bonferroni’s multiple comparison test. *P < 0.05, **P < 0.01, NS = nonsignificant. **b** Effects of overexpression of SP-1, MZF-1, AP-1, and c-Ets-1 on promoter activity of the 4th fragment of the human *CHOP* promoter. Expression plasmids encoding c-myc-tagged SP-1, MZF-1, AP-1, or c-Ets-1 were co-transfected into MLE12 cells with the 4th fragment-*CHOP*-*Luc*-promoter construct and a vector containing a *β-Galactosidase* reporter gene, followed by tunicamycin (2 μg/ml) or vehicle (0.02% DMSO) treatment 24 h after transfection. After 2 h, *Luciferase* and *β-Galactosidase* assays were performed as described in the “Materials and methods” section. As a negative control, the c-myc-tag empty vector was co-transfected together with the pGL4-Hygro empty vector (EV) and the plasmid containing the *β-Galactosidase* reporter gene. Results are presented as normalized *Luciferase* activity. Means ± SD are shown, from *n* = 3 independent experiments, with analysis by Bonferroni’s multiple comparison test. *P < 0.05, **P < 0.01, ***P < 0.001, NS = nonsignificant

**Fig. 6:**
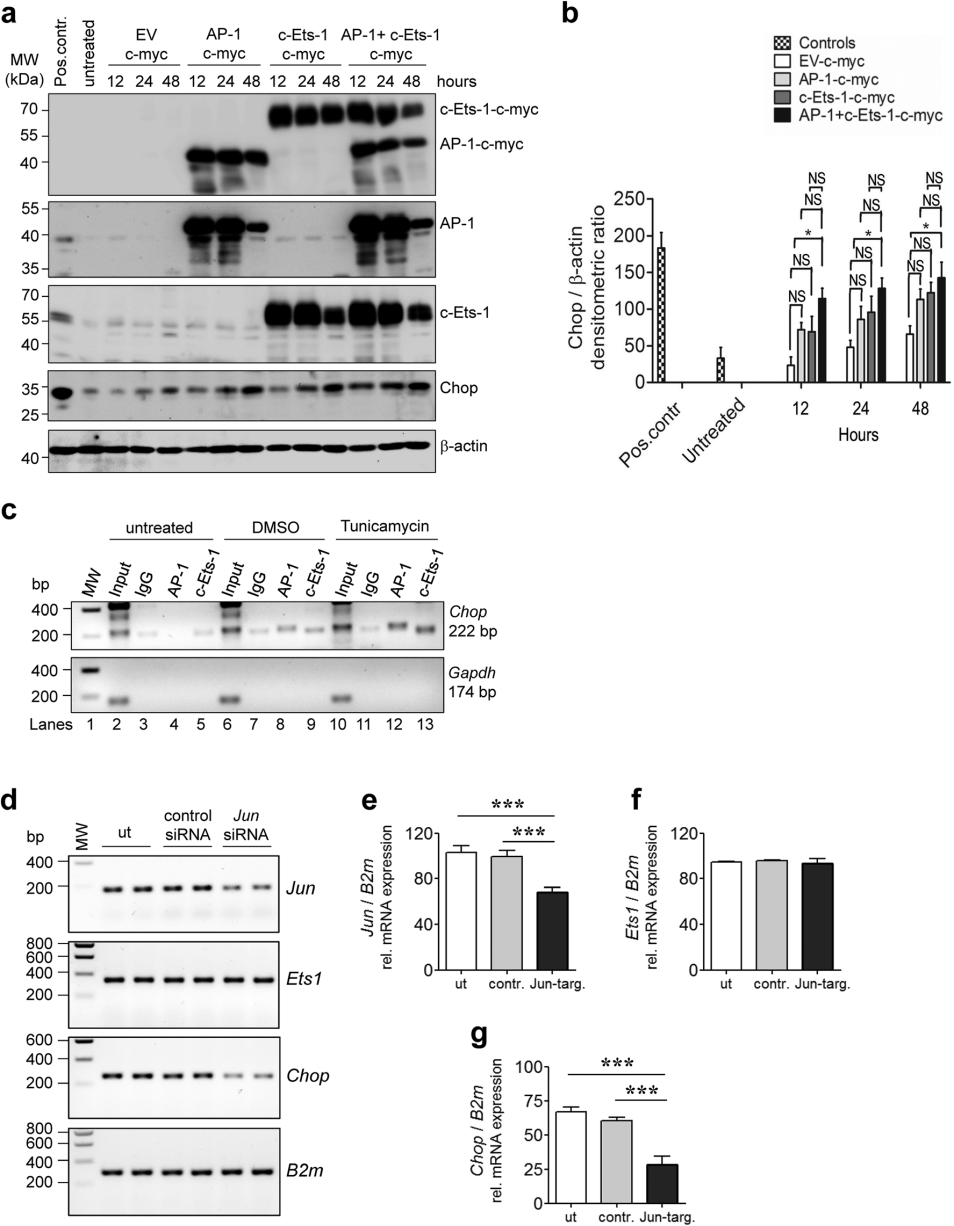
AP-1 and c-Ets-1 regulate the human *CHOP* promoter. **a**, **b** Effect of AP-1 and c-Ets-1 overexpression on Chop expression. MLE12 cells were transfected with AP-1-c-myc, c-Ets-1-c-myc expression plasmid or both for indicated time points, followed by immunoblot analysis for Chop. **b** The protein level for Chop was densitometrically quantified by using ImageJ software. For normalization, β-actin expression was used as a control. Data are expressed as means ± SD, from *n* = 3 independent experiments, with analysis by Bonferroni’s multiple comparison test. *P < 0.05, NS = nonsignificant. **c** ChIP assays were done on untreated, DMSO (0.02%) and tunicamycin (2 μg/ml)-treated MLE12 cells. The extracted chromatin was immunoprecipitated with anti-AP-1 or anti-c-Ets-1 antibodies or with a nonspecific IgG. Immunoprecipitated DNA was analyzed by PCR with primers spanning the 4th fragment of the murine *Chop* promoter; the PCR for a *Gapdh* promoter fragment served as negative control. **d**–**g** Effect of *Jun* (AP-1) knockdown on *Chop* expression. MLE12 cells were transfected with 100 nM nontargeting control siRNA, 100 nM *Jun*-targeting siRNA, or left untreated (ut). At 24 h after transfection, cells were analyzed for *Jun* (**d**, **e**), *Ets1* (**d**, **f**), and *Chop* mRNA expression (**d**, **g**) by RT-PCR. *B2m* served as reference gene. Data are expressed as means ± SD, from *n* = 3 independent experiments, with analysis by Bonferroni’s multiple comparison test. ***P < 0.001

**Fig. 7:**
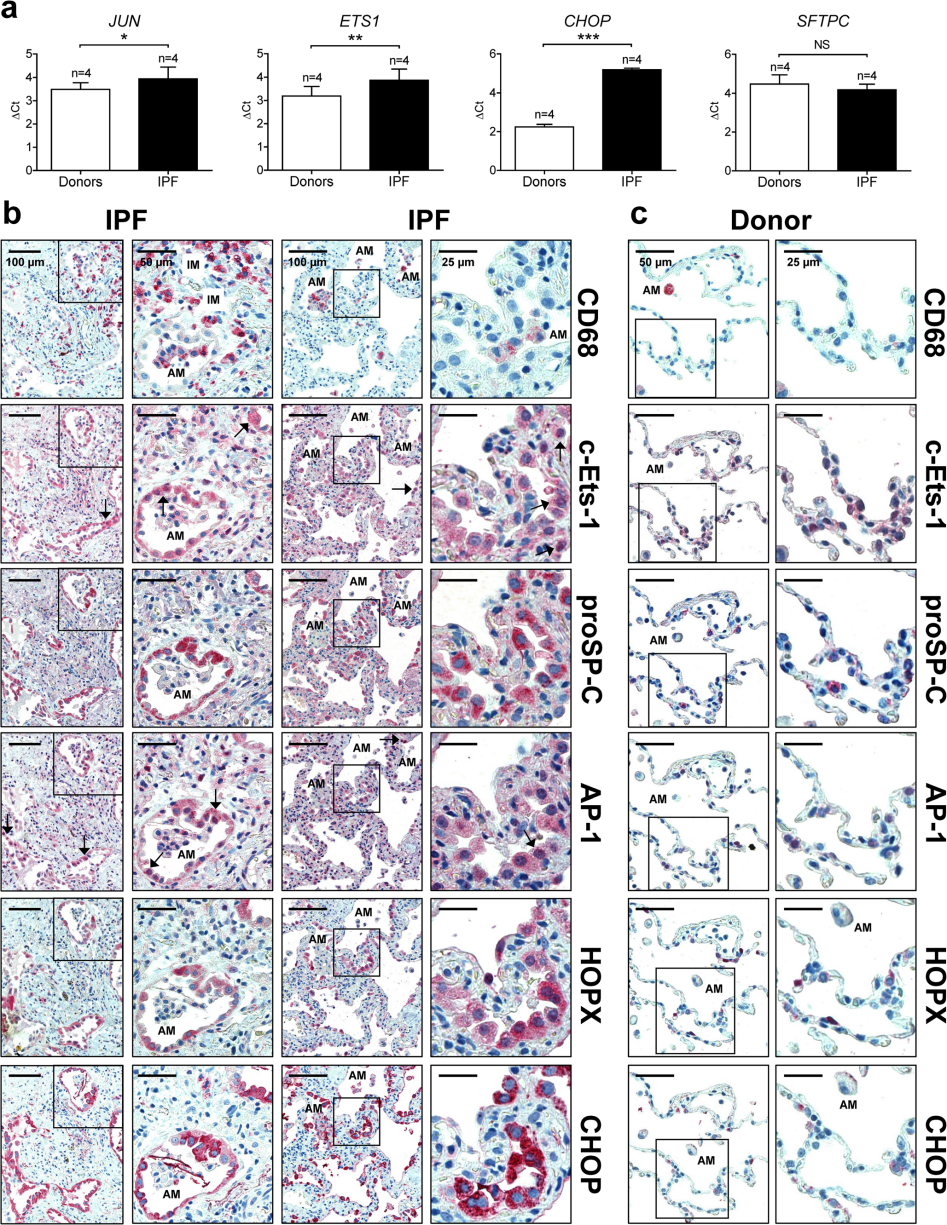
AP-1 and c-Ets-1 are induced in AECII from patients with IPF. **a** Gene expression analysis via qPCR analysis for *JUN*, *ETS1*, *CHOP*, and *SFTPC* in human IPF (*n* = 4) and healthy donor lung tissues (*n* = 4), each done in triplicate. The *ACTB* gene was used as reference gene. Means ± SD are shown, with analysis by unpaired Student’s *t* test. *P < 0.05, **P < 0.01, ***P < 0.001, NS = nonsignificant. **b**, **c** Immunohistochemical staining of serial sections of IPF (**b**) and normal donor lung tissues (**c**) for CD68 (macrophage marker), c-Ets-1, proSP-C (AECII-marker), AP-1, HOPX (AECI-and AECII marker), and CHOP. (**b**) In IPF, proSP-C expressing AECII indicated robust overexpression of c-Ets-1 and AP-1 in the nucleus and cytoplasm (indicated by arrows), and co-localized with induced CHOP expression. CD68-positive interstitial (IM) and alveolar macrophages (AM) of IPF lungs indicated faint or moderate expression of c-Ets-1 and AP-1 and no notable expression of CHOP. **c** In normal donor lungs, AP-1 and c-Ets-1 were expressed at low basal or moderate level in AEC and AM. Minimal or no immunostaining for CHOP was observed in any cells of donor lungs. Results are representative for *n* = 6 IPF patients and *n* = 3 organ donors. Further IHC and IF images can be found in Online Resource 2, Figs. S8–S10

The collaborative action of AP-1 and c-Ets-1 on the human *CHOP* promoter was then confirmed by different, complementary approaches: *first*, by the decreased *CHOP* promoter activity in response to site-directed mutagenesis of the AP-1 and/or c-Ets-1 TF binding sites in the 4th fragment, thereby indicating the most severe impairment in *Luciferase* activity when both AP-1 and c-Ets-1 DNA binding sites were mutated (Fig. 5a); *second*, by the increased promoter activity upon combined versus single overexpression of AP-1 and c-Ets-1 together with the 4th promoter fragment (Fig. 5b); *third*, by the stronger induction of endogenous Chop protein expression upon the combined overexpression of AP-1 and c-Ets-1 compared to the single overexpressions (Fig. 6a, b), even in the absence of ER-stress-inducing agents; *fourth*, by the ChIP analysis of AP-1 and c-Ets-1 binding to the 4th fragment of the murine *Chop* promoter under ER stress conditions (Fig. 6c). Further, we observed that RNAi-mediated silencing of *Jun* (AP-1) resulted in significant reduction of endogenous *Chop* mRNA level (Fig. 6d–g). Together these experiments in MLE12 cells suggest that simultaneous binding of AP-1 and c-Ets-1 is important for the regulation of *Chop*/*CHOP* gene activity in vitro. As CHOP and the other UPR compounds are reported to be highly upregulated in AECII in IPF lungs [8–10], we investigated the expression of AP-1 and c-Ets-1 in IPF versus donor lungs. Indeed, *JUN*, *ETS1*, and *CHOP* expression were significantly upregulated on mRNA level in IPF compared to donor lungs (Fig. 7a). By immunohistochemistry and immunofluorescence, we observed an accumulation of both AP-1 and c-Ets-1 together with CHOP predominantly in proSP-C-expressing AECII of IPF lung tissues, as compared to donor lungs. These showed low to moderate expression of AP-1/c-Ets-1 and minimal immunostaining for CHOP in AECII (Fig. 7b, c, Online Resource 2, Figs. S8–S10). Importantly, AP-1, c-Ets-1, and CHOP were not pronounced in interstitial and alveolar macrophages of IPF lungs, as compared to IPF-AECII. Alveolar macrophages of donor lungs showed low to moderate expression of AP-1 and c-Ets-1 and no immunostaining for CHOP (Fig. 7b, c, Online Resource 2, Figs. S8–S10). In summary, these results suggest that AP-1 and c-Ets-1 are involved in ER-stress-induced *CHOP* regulation in the AECII during fibrosis development.

### Epithelial Chop overexpression in vitro results in apoptosis and fibroproliferative signaling

**Fig. 8:**
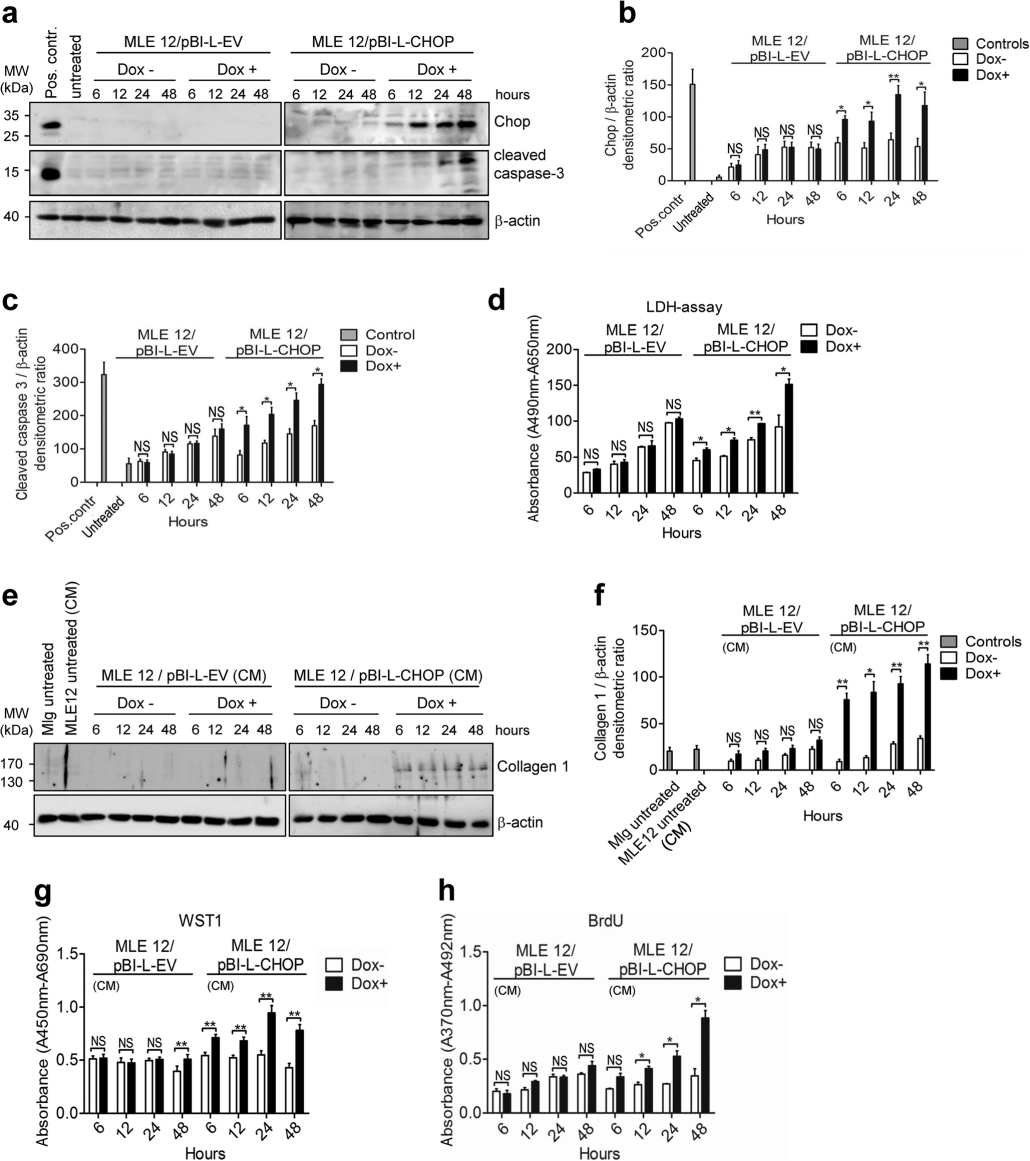
Effect of Chop overexpression on apoptosis of epithelial cells and lung fibroblast phenotype in vitro. **a**–**c** Stably transfected MLE12/pBI-L-EV (empty vector control) and MLE12/pBI-L-CHOP cells were treated with 1 μg/ml doxycycline (Dox+, for transgene induction) or left untreated (Dox−, control) for indicated time points, followed by quantitative immunoblot analyses for Chop and cleaved caspase-3. The protein levels for Chop (**b**) and cleaved caspase-3 (**c**) were densitometrically quantified by using ImageJ software. For normalization, β-actin expression was used as a control. Data information: Untreated = untreated MLE12 cells; Pos. contr. = positive control, MLE12 cells treated either with 1 μg/ml tunicamycin (24 h) or 1 μM/ml staurosporine (8 h). **d** LDH assay quantifying MLE12 cell death in response to doxycycline (Dox+)-induced conditional Chop overexpression in MLE12/pBI-L-CHOP cells, as compared to (Dox+)-treated MLE12/pBI-L-EV cells and (Dox−) conditions. **e**, **f** Conditioned medium (CM) from stably transfected MLE12/pBI-L-EV (empty vector control) and MLE12/pBI-L-CHOP cells in the presence (Dox+) or absence of doxycycline (Dox−) was applied to cultured Mlg lung fibroblasts, followed by reincubation for 24 h and assessment of collagen synthesis in fibroblasts by quantitative immunoblotting for collagen 1. The protein levels for collagen 1 (**f**) were densitometrically quantified by using ImageJ software. For normalization, β-actin expression was used as a control. **g**, **h** Proliferation of Mlg lung fibroblasts by WST-1 assay (**g**) or BrdU incorporation (**h**) in response to culture with conditioned media (CM) of stably transfected MLE12/pBI-L-EV and MLE12/pBI-L-CHOP cells in the presence (Dox+) or absence of doxycycline (Dox−). All data are expressed as means ± SD, from *n* = 3 independent experiments, with analysis by unpaired Student’s *t* test. *P < 0.05, **P < 0.01, NS = nonsignificant

To investigate the impact of Chop on apoptosis of AECII, we overexpressed it conditionally in stably transfected epithelial MLE12 cells, by using an inducible “Tetracycline-On” vector system. As compared to stably transfected MLE12/pBI-L empty vector cells, stably transfected MLE12/pBI-L-CHOP cell lines conditionally expressed Chop in response to doxycycline (Dox+) treatment (Fig. 8a, b). Increased levels of cleaved caspase-3 were already detected 6 h after Chop overexpression in MLE12, thus indicating a rapid, apoptotic response of the AECII in response to Chop induction (Fig. 8a, c). Accordingly, extracellular lactate dehydrogenase (LDH) was found to be significantly increased 6 h after Chop overexpression (Fig. 8d). The proapoptotic signaling response elicited by Chop overexpression was also verified by synchronous, upregulated expression of Chop targets Gadd34 (growth arrest and DNA damage-34) [20] and Dr5 [15, 18] in stably transfected MLE12/pBI-L-CHOP cells after (Dox+) treatment, as compared to noninduced (Dox−) conditions and MLE12/pBI-L empty vector cells (Online Resource 2, Fig. S11a–c).

Taking into consideration that intensive fibroblast proliferation and collagen deposition is a hallmark of lung fibrosis, we investigated the profibrotic/proproliferative signal of conditioned media (CM) from Chop expressing MLE12 on cultured Mlg lung fibroblasts. Conditioned media from Dox+-treated MLE12 cells overexpressing Chop, but not from (Dox−) cells or empty vector (EV) controls, induced collagen 1 protein expression and mouse lung fibroblast proliferation, as shown by immunoblotting (Fig. 8e, f) and WST1 assay or BrdU incorporation (Fig. 8g, h), respectively. Moreover, overexpression of Chop in primary mouse AECII by adenoviral gene transfer also resulted in AECII apoptosis and consecutive profibrotic responses in lung fibroblasts, as compared to adenoviral empty-vector-infected cells (Online Resource 2, Fig. S12a–h).

## Discussion

The proapoptotic, ER-stress-related transcription factor CHOP is present at low levels under physiological conditions but is highly upregulated in response to ER stress [15, 21], hypoxia [26, 27], or DNA damage [28], which may also lead to misfolded protein accumulations and UPR activation [26–28]. CHOP expression is mainly regulated at the transcriptional level [29]. In our study, we could show that, besides the previously reported ERSE and AARE elements, two adjacent TF binding sites, namely the AP-1 (bases − 246 to − 240) and c-Ets-1 (bases − 205 to − 199) binding sites, act in concert and are essential for ER-stress-induced *CHOP* gene activation. In agreement, we also observed that combined overexpression of AP-1 and c-Ets-1 in MLE12 cells alone in the absence of ER stress inducers was sufficient to induce Chop protein expression.

The transcriptional control of the *CHOP* promoter via AP-1 and c-Ets-1 is interesting, because singular or combined overexpression of the main ER stress transducers Atf4, p50Atf6, and sXbp1 was not sufficient to induce *Chop* mRNA in our experiments. Also, these three highly conserved TFs failed to induce substantial *Ets1* and *Jun* expression in MLE12 cells in comparison to thapsigargin treatment, as shown by our transcriptome analysis. The existence of additional AP-1 and c-Ets-1 regulated elements in the *CHOP* promoter thus offers a plausible explanation for the seemingly negligible *Chop* expression in response to overexpression of “conventional” UPR transducers alone.

It had been previously suggested that the AP-1 binding site of the *CHOP* promoter contributes to *CHOP* gene activation in response to oxidative stress [30], brefeldin A treatment [31], and in mitochondrial unfolded protein response [32]. Our data suggest that the *CHOP* transcriptional activity is, next to the conventional signaling via AARE and ERSE, regulated by the concomitant activation of the AP-1 and c-Ets-1 elements in response to TM. In agreement, MLE12 cells as well as primary AECII induced and overexpressed AP-1 and c-Ets-1 in response to TM, concomitantly with the upregulation of the ER stress markers Atf4, p50Atf6, sXbp1, and Chop.

Others reported that *ETS1*/c-Ets-1 upregulation upon ER stress (induced by TM) was mediated by the IRE1α/XBP1 branch and inhibited in cell lines deficient in IRE1α or XBP1 [33], suggesting the importance of the spliced XBP1-TF in activating *ETS1* expression. This is not necessarily in contradiction to the lack of an increase in *Ets1* in AECII-like MLE12 cells in response to overexpression of sXbp1, as observed in our study. Spliced XBP1 may require additional factors to activate *ETS1* expression.

C-Ets-1 is a member of the E26 transformation-specific (Ets) transcription factor family. It is involved in oncogenic transformation, angiogenesis, differentiation, and apoptosis [34–36]. C-Ets-1 can be activated by extracellular signal-regulated kinase 1/2 (ERK1/2) [35, 36]. In mice with diabetic cardiomyopathy, high mobility group box 1 protein (HMGB1) mediates hyperglycemia-induced cardiomyocyte apoptosis via ERK/c-Ets-1 signaling pathway [36]. The proapoptotic function of c-Ets-1 is also supported by studies from Teruyama and coworkers, where overexpression of c-Ets-1 in human umbilical vein endothelial cells (HUVECs) induced apoptosis under serum-deprived conditions, which could be blocked by caspase inhibitors [37]. C-Ets-1 upregulated various proapoptotic genes, including *BID* (BH3-interacting domain death agonist) and *CASP4* (caspase-4), and downregulated anti-apoptotic genes such as *DIAP2* (death-associated inhibitor of apoptosis-2) in serum-deprived HUVECs [37]. However, it is not clear from this study whether c-Ets-1 directly activates these genes [37]. Additionally, caspase-1 (*CASP1*) has been reported as a direct target gene of c-Ets-1 in cancer cells [38]. C-Ets-1 also induces *CTGF* (connective tissue growth factor) expression in dermal and cardiac fibroblasts, which is consistent with a role in tissue fibrotic remodeling [39, 40]. These observations indicate that c-Ets-1 signaling is not always proapoptotic and appears to be cell context specific.

Atanelishvili et al. [41] showed that primary AECII or A549 activate CHOP expression through a c-Ets-1-dependent pathway in response to thrombin exposure, which was associated with consequent apoptosis in both cell types. On the contrary, thrombin decreased tunicamycin-induced CHOP expression in lung fibroblasts through a Myc-dependent mechanism and protected these cells from apoptosis [41]. Thus, regulation of CHOP expression by thrombin may contribute to persistent fibroproliferation in fibrotic lung diseases, in which thrombin activates c-Ets-1 and CHOP driving AECII apoptosis while promoting survival of lung fibroblasts.

Similar to c-Ets-1, dual roles have also been reported for AP-1/c-Jun. It has been demonstrated in various kidney fibrosis models that AP-1 promotes transcription of profibrotic genes (e.g., *COL1A1*, *FN*) in fibroblastic cell populations [42]. Wernig et al. have recently described that systemic ubiquitous induction of c-Jun in mice resulted in the development of fibrosis in various organs, including the lung, and that the proproliferative and profibrotic effects of c-Jun were mainly restricted to fibroblastic cell populations [43]. Further, they observed AP-1/c-Jun expression in myofibroblasts as well as in cytokeratin-7 (KRT7)-positive epithelial cells of patients with IPF. KRT7 is a marker for epithelial cells, including AECI+II, Club cells, and ciliated bronchial cells. Because AECII apoptosis is a hallmark of IPF, AP-1 induction may be associated with programmed cell death in the IPF-AECII, but with increased proliferation and ECM production in IPF fibroblasts.

In line with the CHOP induction and apoptotic cell death in alveolar epithelium of IPF lungs [8], we found both AP-1 and c-Ets-1 to be overexpressed in AECII of IPF lungs, but not in donor AECIIs. Further, we observed only faint to moderate immunostaining for AP-1 and c-Ets-1 in the interstitium of IPF lungs, with no immunoreactivity for CHOP. We, therefore, suggest that AP-1 and c-Ets-1 are upregulated in IPF-AECII upon injury caused by maladaptive ER stress in order to promote expression of CHOP and AECII apoptosis.

We showed that CHOP overexpression in AECII-like MLE12 cells induced Gadd34 and Dr5, which have been reported to execute Chop-mediated apoptosis [15, 18, 20]. In addition, CHOP has been described as a multifunctional transcription factor, being involved in induction of many other proapoptotic genes, such as *ATF5* (activating transcription factor-5) [44], *TRB3* (tribbles homolog 3) [45], or *CASP11* (caspase-11) [46, 47], but also of inflammatory cytokines *IL1B* (interleukin-1β) [48] and *IL6* (interleukin-6) [49], thereby perpetuating cell injury and death.

The molecular mechanisms leading to fibrogenesis in response to AECII death are still incompletely resolved, but may be based on soluble mediators released from injured AECIIs such as profibrotic cytokines, growth factors, or other mediators [7, 50]. Supernatants of Chop-overexpressing AECII and MLE12 cells on cultured lung fibroblasts in vitro enhanced the proliferation and collagen production in fibroblasts. Thus, our results suggest that overexpression of Chop in AECII alone without any other triggers is capable to induce both AECII apoptosis and consecutive profibrotic responses in lung fibroblasts.

ER-stress-mediated cell death involving Chop-induced apoptosis has been documented in various pathological conditions, including inflammatory bowel disease [46], myocardial and renal ischemia–reperfusion injury [51, 52], type 2 diabetes [15, 53], steatohepatitis [54], and organ fibrosis involving the liver, kidney, and lung [26, 54–57]. Accordingly, deletion of the *Chop*/*CHOP* gene in a variety of cell lines in vitro has been shown to protect from ER-stress-induced apoptosis [15, 16], and full Chop deficiency in homozygous *Chop*^(−/−)^ knockout (ko) mice has been shown to prevent or attenuate the abovementioned disorders [26, 46, 51–57].

Moreover, development of lung fibrosis in response to bleomycin, which is accompanied by UPR activation, nuclear Chop induction, and apoptosis in the AECII of treated mice [26, 56], has been observed by three independent groups to be abolished in homozygous *Chop*^(−/−)^ ko mice [26, 56, 57]. However, one contrary report also exists by Ayaub and coworkers, describing that full blockade of Chop by the use of *Chop*^(−/−)^ ko mice did not protect from bleomycin-induced fibrosis, and even *wild-type* mice were better protected [58]. The authors described that Chop-mediated macrophage apoptosis is protective toward bleomycin-induced fibrosis in *wild-type* mice. In contrast, *Chop*^(−/−)^ ko mice were unable to activate ER-stress-induced apoptosis in macrophages in response to bleomycin treatment, which led to increased inflammation, interstitial fibrosis, and abnormally high levels of (nonapoptotic) macrophages, especially of the profibrotic M2 phenotype, both in the BALF and in the lung parenchyma of the knockout mice [58]. However, these results stand in marked contrast with the work of Yao and coworkers describing that Chop deficiency in *Chop*^(−/−)^ ko mice protected against bleomycin-induced lung injury and fibrosis, paradoxically by attenuating M2 macrophage production [57]. In addition, Burman et al. have recently reported that *Chop*^(−/−)^ ko mice were even protected in the repetitive intratracheal bleomycin-fibrosis model, and this was due to significantly reduced AECII apoptosis [26]. Further, the authors described that relevant differences were not detected in the numbers of interstitial and alveolar macrophages in the lungs of *wild-type* versus *Chop*^(−/−)^ ko mice. However, *Chop*^(−/−)^ ko mice were not protected from lung fibrosis in response to single-dose intratracheal bleomycin; but the explanation for this phenomenon is very different from that by Ayaub et al. Burman and colleagues observed that single intratracheal bleomycin was not sufficient to induce robust Chop upregulation in *wild-type* mice thus offering a potential reason why *Chop* deletion was not protective in this model [26].

In regard to human IPF, we could show that CHOP and its regulators AP-1 and c-Ets-1 do not play a pivotal role in macrophages, whereas they are dominant in the IPF-AECII. Taken together, our and the data from other groups indicate that the pathomechanistic role of CHOP is AECII specific under conditions of lung fibrosis.

In summary, our study suggests that AP-1 and c-Ets-1 represent important transcriptional regulators of the *CHOP* gene, possibly explaining the differences between adaptive ER stress caused by activation of the conventional UPR branches PERK, ATF6, and IRE1α/XBP1, without induction of CHOP, and the “maladaptive” proapoptotic ER stress characterized by CHOP induction through additional activation of the AP-1 and c-Ets-1 TF binding sites within the *CHOP* promoter. We propose that *CHOP* activation by AP-1 and c-Ets-1 plays a key role in AECII maladaptive ER stress responses and consecutive fibrosis, offering new therapeutic prospects in IPF.

## Electronic supplementary material

ESM 1 (PDF 509 kb)

ESM 2 (PDF 6820 kb)
