# Supplementary material for: Regulation and role of the ER stress transcription factor CHOP in alveolar epithelial type-II cells
Source: J Mol Med (Berl). 2019 Apr 26;97(7):973–90. doi: 10.1007/s00109-019-01787-9 (PMC6581940; doi:10.1007/s00109-019-01787-9)
Supplement: Supplementary file 1 — (PDF 509 kb) [file 109_2019_1787_MOESM1_ESM.pdf]

Regulation and role of the ER-stress transcription factor CHOP in alveolar epithelial type-II cells

Supplementary Material and Methods
Supplementary Tables S1-S6

Oleksiy Klymenko[1,2], Martin Huehn[1,2], Jochen Wilhelm[1,2], Roxana Wasnick[1,2], Irina Shalashova[1,2], Clemens Ruppert[1,2,3], Ingrid Henneke[1,2], Stefanie Hezel[1,2], Katharina Guenther[1,2], Poornima Mahavadi[1,2], Christos Samakovlis[1,2,3,4], Werner Seeger[1,2,3,5], Andreas Guenther[1,2,3,6,7]*‡, Martina Korfei[1,2‡].

‡These authors contributed equally to this work.

Affiliations:

[1]Department of Internal Medicine, Justus-Liebig-University Giessen, D-35392 Giessen, Germany.

[2]Universities of Giessen and Marburg Lung Center (UGMLC), Member of the German Center for Lung Research (DZL), D-35392 Giessen, Germany.

[3]Excellence Cluster Cardiopulmonary System (ECCPS), D-35392 Giessen, Germany.

[4]Department of Molecular Biosciences, The Wenner-Gren Institute, Stockholm University, SE-106 91 Stockholm, Sweden.

[5]Max-Planck-Institute for Heart and Lung Research, Department of Lung Development and Remodeling, D-61231 Bad Nauheim, Germany.

[6]European IPF Network and European IPF Registry.

[7]Agaplesion Lung Clinic Waldhof-Elgershausen, D-35753 Greifenstein, Germany.

***Corresponding Author**
Andreas Guenther, M.D.
Department of Internal Medicine
Justus-Liebig-University Giessen
Klinikstrasse 36
D-35392 Giessen, Germany
E-mail: Andreas.Guenther@innere.med.uni-giessen.de
Tel.: +49 641 98542502; Fax: +49 641 98542508

Supplementary Material and Methods

Cell culture experiments

MLE12 cells (ATCC® CRL-2110™) were maintained in DMEM/F12 medium (Gibco) supplemented with 2% (v/v) fetal bovine serum (FBS, PAA), 10 nM hydrocortisone (Gibco), 10 nM hydro-beta-estradiole (Fischer ICN), 5 µg/ml insulin (PAN Biotech), 10 µg/ml transferrin (PAN Biotech), 30 nM Na-selenite (PAN Biotech), 10 mM HEPES (Sigma), 2 mM L-Glutamine (Gibco) and 1% (v/v) penicillin/streptomycin (Gibco). A549 cells (ATCC® CCL-185™) were maintained in DMEM/F12 medium (Gibco) supplemented with 10% (v/v) FBS (PAA), 1% (v/v) vitamins (Gibco), 1% (v/v) non-essential amino acids (Gibco) and 1% (v/v) penicillin/streptomycin (Gibco). HEK293T cells (ATCC® CRL-1573™) and murine fibroblast cell line Mlg (ATCC® CCL-206™) were maintained in DMEM medium (Gibco) supplemented with 10% (v/v) FBS (PAA), 2 mM L-Glutamine (Gibco) and 1% (v/v) penicillin/streptomycin (Gibco). All cell lines were grown in 10 cm^2 tissue culture plates at 37°C, 5% CO_2. Cells were seeded and cultured to 60–80% confluency.

Transfection of MLE12 cells with siRNAs was performed with Dharmafect 1 reagent (Dharmacon) or with Lipofectamine® RNAiMAX-reagent (Invitrogen) according to the manufacturer´s instructions. MLE12 cells were transfected with 0.1 µM siRNAs targeting either *Atf4* (siGENOME siRNA D-042737-01), *Atf6* (siGENOME siRNA D-044894-03), *Jun* (ON-TARGETplus Mouse *Jun* (16476) siRNA, SMART pool, 043776-00-0005) or with non-targeting siRNA (siGENOME non-Targeting siRNA-1); and siRNA-transfected cells were harvested for mRNA- and protein isolation 24h or 48h after transfection. All siRNAs were from Dharmacon.

For induction of ER-stress or apoptosis, cells were treated with vehicle [0.02% (v/v) dimethyl sulfoxide, DMSO], and either with 2 µg/ml tunicamycin (TM) (Calbiochem) for 15 min, 30 min, 1h, 2h, 5h, or with 1 µg/ml TM for 24h, or with 1 µM/ml staurosporine (Calbiochem) for 8h. For microarray experiments, cells were treated for induction of ER-stress with 1 µM/ml thapsigargin (TG) (Calbiochem).

Prior to the use in these experiments, all mentioned cell lines were tested for mycoplasma contamination by using commercially available kits (Thermo Fisher Scientific).

Microarray analysis

MLE12 cells were transfected with expression plasmids pIRES2dsRed2 containing *Atf4*, *Atf6* or spliced *Xbp1* cDNA. Transfection with empty-vector served as control. Cells were lysed for RNA isolation 22, 44 and 66 hours after transfection. As a positive control for strong ER-stress induction, cells transfected with empty-vector were treated with 1 µM/ml thapsigargin (TG) (Calbiochem) 22 hours after transfection, followed by harvesting of cells for RNA isolation 22 hours later (n=6 for each of these conditions). A second experiment was designed to assess gene regulation induced by early ER-stress. ER-stress was induced by 1 µM/ml TG, followed by harvesting of cells for RNA isolation after 0.5, 1 and 6h (n=4 for each condition, n=12 for control = buffer treatment). Total RNA was isolated using the RNeasy Plus Mini Kit (Qiagen). Purified total RNA was amplified and Cy-dye-labeled using the dual-color QuickAmp labeling kit (Agilent, P/N 5190-0444) following the kit instructions. Per reaction, 1 µg of total RNA was used. The samples were labeled with either Cy3 or Cy5 to match a balanced dye-swap design. Cy3- and Cy5-labeled RNAs were hybridized overnight to 4×44K 60mer oligonucleotide spotted microarray slides (Mouse Whole Genome 4×44K, Agilent Technologies, P/N G4122F).

Hybridization and subsequent washing and drying of the slides were performed following the Agilent hybridization protocol. The dried slides were scanned using the GenePix 4100A scanner (Axon Instruments, Downingtown, PA) at a resolution of 10 pixels/µm. Image analysis was performed with GenePix Pro 5.1 software and calculated values for all spots were saved as GenePix results files. Data were evaluated using the R software [S1] and the limma package [S2] from BioConductor [S3]. Annotations were updated from Ensemble using biomart (http://www.biomart.org). The intensity values were quantile normalized before averaging [S4]. Genes were ranked for differential expression using a moderated t-statistic. Candidate lists were created by adjusting the false-discovery rate to 5%. Pathway analyses were done using the function geneSetTest from limma on the ranks of the t values.

Microarray data have been uploaded to GEO on 09/23/2016. The accession number is **GSE87298**. To access the data, use the following link:

https://www.ncbi.nlm.nih.gov/geo/query/acc.cgi?token=efqzsaymdzkvbun&acc=GSE87298

Luciferase assay

Cells in 12-well plates were transfected with 900 ng of generated *CHOP-Luciferase* promoter constructs and 100 ng of β-Galactosidase vector. After 24 hours, cells were treated with vehicle [0.02% (v/v) dimethyl sulfoxide, DMSO] or 2µg/ml tunicamycin for 2h. Thereafter, Luciferase assay (Promega) and β-Galactosidase assay (Promega) were performed according to the manufacturer's instruction. Briefly, cells were washed with ice-cold PBS and Reporter lysis buffer (Promega) was applied. For cell lysis, one cycle of freezing at -80°C overnight and thawing on ice was performed. To measure *Luciferase* and *β-Galactosidase* activity, 20 µl and 50 µl of clear extract were used, respectively. *Luciferase* activity was measured by a

luminometer (SpectraFluor Plus, Tecan); and *β-Galactosidase* activity was determined by absorbance at 420 nm using the spectrophotometer (SpectraFluor Plus, Tecan). Results of *Luciferase* activity were normalized against *β-Galactosidase* activity.

Stably-transfected epithelial cell lines

Two sets of plasmids were used for conditional and stable overexpression of Chop. The regulatory-plasmid pTet-On (Clontech), and the response-plasmid pBI-L (Clontech), which allow the expression of the gene of interest and luciferase from a bidirectional tetracycline responsive promoter, were co-transfected in MLE12 cells. Prior to transfection, pTet-On vector was *linearized* with *HindIII* (New England BioLabs). MLE12 cells were then transfected with 2 µg of the *linearized* pTet-On. 32h post-transfection, growth DMEM/F12-medium was replaced with medium containing 500 µg/ml of G418/Geneticin (Roche), and cells were re-incubated for additional 48h for selection of clones. Stably-transfected cells were established by limited-dilution-method. Thereafter, MLE12/pTet-On stably-transfected cells were co-transfected either with pBI-L-empty-vector or the pBI-L-CHOP together with the *linear hygromycin marker* (Clontech). Before transfection, these plasmids were *linearized* with *AatII* (New England BioLabs). Transfection and selection of clones were performed as described above. Thereafter, multiple cell clones of stably co-transfected MLE12/pTet-On cells were selected by adding 100 µg/ml of G418 and 100 µg/ml of hygromycin (InvivoGen) to the medium. In order to identify MLE12/pBI-L-CHOP or MLE12/pBI-L-EV cells, in which *Chop*- or *Luciferase*-expression was effectively regulated by doxycycline, all antibiotic resistant clones were treated with 1 µg/ml of doxycycline (Roche) or left untreated. Cells were harvested 24h after treatment and the activity

of the reporter gene *Luciferase* was detected using the Luciferase Reporter Assay System (Promega).

Alveolar type II cells (AECII) culture

Primary murine AECII were isolated from the lungs of C57BL/6J mice as described [S5, S6]. The AECII were plated on wells of a 6-well-plate (1×10^6/well) coated with Matrigel (BD Bioscience, Germany). Cells were cultured in BEGM medium (Lonza) supplemented with 5% (v/v) CS-FBS (HyClone) and 10 ng/ml hKGF (PreproTech). In this study, AECII cells were cultured up to 4 days. All cultures were maintained in humidified atmosphere with 5% CO_2 at 37°C. For analysis of ER-stress-response, AECII in 6-well-plates were treated with vehicle [0.02% (v/v) dimethyl sulfoxide, DMSO] or with 2 µg/ml tunicamycin (TM) (Calbiochem) for 15 min, 30 min, 1h, 2h and 5h, or left untreated. After indicated time-points, AECII cells were harvested, followed by protein isolation.

Adenovirus production and infection of primary AECII with adenoviral vectors

Generation of adenoviruses was performed using two adenoviral vectors. The first adenoviral plasmid was pAdEasy-1 (Addgene), the second vector was pAdTrack-CMV (Addgene), which is used for the expression of transgenes. Recombinant adenoviruses carrying *Chop*-cDNA, or GFP were generated by homologous recombination between the expression cosmid cassette (pAdTrack) and parental virus genome (pAdEasy-1) in *E. coli* BJ5183 cells (Agilent Technologies). Before co-transformation, pAdTrack-CMV-EV and pAdTrack-CMV-CHOP were linearized with *PmeI* (New England BioLabs). The adenoviral constructs were digested with *PacI* (New England BioLabs) and transfected into the permissive HEK293T cell line to generate

recombinant Adeno-Chop (Ad-Chop) or Adeno-empty-vector, containing GFP (Ad-EV) viral particles. The viral titer was determined by the ′tissue culture infectious dose-50′-method. Primary AECIIs were infected with adenoviruses at a 10× multiplicity of infection. Infected AECIIs were cultured for 4 days, and at indicated time-points cells were harvested for mRNA- and protein isolation.

Real-time PCR analysis (quantitative PCR)

RNA was extracted from 1×10^6 cells or 30 mg of lung tissue using RNeasy Plus Mini Kit (Qiagen). Two µg of RNA sample was reverse-transcribed to cDNA using Omniscript Reverse Transcription Kit (Qiagen) and Oligo-dT primers (Applied Biosystem). 25-50ng of cDNA was then subjected to a SYBR-green-based real-time PCR amplification-system by using gene-specific primers. The real-time PCR was performed using the Mx3000P Real-Time PCR system with MxPro v4.10 software (Stratagene), according to manufacturer's instructions. Relative mRNA expression levels were calculated using the ΔCT and 2^(-ΔΔCT) method [S7] where ΔCT is defined by $CT_{\text{gene of interest}} - CT_{\text{housekeeping gene}}$. The *ACTB*/*Actb* gene was used as reference gene and the primers used for the real-time RT-PCR analyses are listed in Table S5. With the exception of spliced *Xbp1* [S8], all other gene-specific primers were designed by the authors.

Western blot

Protein extracts from cells were prepared using a lysis buffer containing 50 mM Tris-HCl/pH 8.0, 5 mM EDTA, 150 mM NaCl, 1% (v/v) Triton-X-100, 0.5% (w/v) Na-deoxycholate and 1 mM PMSF. Protein extracts were subjected for separation to SDS-PAGE, followed by transfer

onto PVDF membranes (Roth) in a semi-dry blotting chamber according to the manufacturer´s protocol (Bio-Rad). Obtained immunoblots were then blocked by incubating at room temperature (RT) for 1h in blocking buffer [1 × tris-buffered saline (TBS; 50 mM tris-HCl, pH 7.5, 50 mM NaCl) containing 5% (w/v) nonfat dried milk and 0.1% (v/v) tween 20], followed by immunostaining for the proteins of interest. The primary antibodies used for western blotting are listed: anti-c-myc-tag (1:60000, Abcam, ab9106), anti-Gadd153 (1:1000, Santa Cruz, sc-575), anti-Chop (1:300, CST, #5554), anti-c-Jun/AP-1 (1:1000, Santa Cruz, sc-44), anti-c-Jun/AP-1 (1:300, abcam, ab31419), anti-ATF4 (1:6000, Aviva Systems Biology, #37017_P050), anti-ATF6 (1:1000, Abcam, ab37149), anti-c-Ets-1 (1:1000, Santa Cruz, sc-350), anti-c-Ets-1 (1:300, abcam, ab26096), anti-XBP-1 (1:300, abcam, ab198999), anti-cleaved caspase-3 (1:500, Cell Signalling, #9664), anti-DR5 (1:500, Abcam, ab47179), anti-GADD34 (1:300, Santa Cruz, sc-825), anti-Collagen-I (1:500, Rockland, #600-401-103), anti-GAPDH (1:2000, abcam, ab181602) and anti-β-actin (1:10000, Abcam, ab8227). The blots were then incubated with horseradish peroxidase-conjugated secondary swine anti-rabbit IgG antibodies (DakoCytomation, diluted 1:1000 in blocking buffer) for 2 hours at RT. Blot membranes were developed with the Immobilon Western Chemiluminescent HRP substrate (Millipore), and emitted signals were detected with a chemiluminescence imager (Intas ChemoStar, Intas). Thereafter, blots were stripped for 1h using Stripping Buffer (Thermofisher Scientific), followed by re-probing the blots with antibodies against the loading controls Gapdh or β-actin. For quantification, band intensities were quantified by densitometry using ImageJ software (Version 1.46r, NIH). The band densities were normalized to Gapdh/β-actin as indicated in figures.

ChIP analysis

MLE12 *cells* (1×10^7) were treated with 0.02% (v/v) DMSO, 2 µg/ml tunicamycin, or left untreated for 2h, followed by crosslinking with 1% (v/v) formaldehyde (Sigma) for 10 min. The crosslinking was stopped by 125 mM glycine (Roche) for 5 min. Cells were then lysed for 10 min with lysis buffer (50 mM Tris-HCl/pH 8.0, 2 mM EDTA, 0.1% (v/v) NP-40, 10% (v/v) Glycerol, 2 mM dithiothreitol). The nuclear fraction was extracted with a buffer containing 50 mM EDTA, 1% (w/v) SDS and 2 mM dithiothreitol. Chromatin was then sonicated to obtain 200–500 bp chromatin-fragments. Sonicated chromatin was diluted 1:9 with 50 mM Tris-HCl/pH 8.0, 5 mM EDTA, 0.5% (v/v) NP-40, and 200 mM NaCl. Chromatin was then incubated with 2 µg of rabbit IgG (Santa Cruz, sc-2027), anti-c-Jun (Santa Cruz, sc-44) or anti-c-Ets-1 (Santa Cruz, sc-350) under gentle shaking overnight, followed by incubation with 30 µl of Protein A-agarose beads conjugated with salmon sperm DNA (Millipore). Thereafter the beads were washed 1 time with a buffer containing 20 mM Tris-HCl/pH 8.0, 2 mM EDTA, 1% (v/v) NP-40, 0.1% (w/v) SDS, 150 mM NaCl, followed by a buffer including 20 mM Tris-HCl/pH 8.0, 2 mM EDTA, 1% (v/v) NP-40, 0.1% (w/v) SDS, 500 mM NaCl, and a buffer with 10 mM Tris-HCl/pH 8.0, 1 mM EDTA, 1% (v/v) NP-40, 1% (w/v) Na-deoxycholate, 250 mM LiCl, and then finally with TE buffer (10 mM Tris-HCl/pH 8.0, 1 mM EDTA). Chromatin was eluted by shaking at 900 rpm with DNA-elution buffer (10 mM Tris-HCl/pH 8.0, 1 mM EDTA, 100 mM $NaHCO_3$, 1% (w/v) SDS) supplemented with 1 mg/ml of RNaseA (Thermo Scientific), for 1 hour at 37°C. Thereafter beads were incubated for 2h at 56°C under shaking at 900 rpm in elution buffer containing 10 mg/ml Proteinase K (Peqlab). The crosslinking was reversed overnight at 65°C after addition of 200 mM NaCl. The DNA was then purified by phenol-chloroform extraction and subjected to PCR analysis using HotStar Taq DNA Polymerase (Qiagen),

employing primers spanning the 4th fragment of the *Chop* promoter. The PCR for a *Gapdh* promoter fragment was employed as a negative control. Primer: *Chop* forward 5'-TCCCCGCCCCCCTTTCCT-3', *Chop* reverse 5'-GCCCCGCCCCGTGCCT-3'; *Gapdh* forward 5'-ATGGTTGCCACTGGGGATCT-3', *Gapdh* reverse 5'-TGCCAAAGCCTAGGGGAAGA-3'. PCR products were analyzed by 1.5% agarose gel electrophoresis.

Proliferation assays

WST-1 (4- [3- (4- iodophenyl)- 2- (4-nitrophenyl)- 2H- 5-tetrazolio]- 1,3-benzene disulphonate) (Roche) is a colorimetric assay that quantifies mitochondrial dehydrogenase activity and thus reflects cell viability. Colorimetric bromodeoxyuridine (BrdU) assay (Roche) is based on incorporation of BrdU into newly synthetized DNA in proliferating cells. WST-1 and BrdU assays were performed according to manufacturer's instructions. Briefly, MLE12/pBI-L-CHOP or MLE12/pBI-L-EV cells were grown in 12-well plates in complete medium containing 100 µg/ml of both G418 and hygromycin. At 60% confluency, cells were treated for the indicated time with 1 µg/ml of doxycyclin (Sigma) or left untreated. Thereafter, supernatants of Chop overexpressing MLE12 cells were used for lung fibroblast-proliferation experiments. Approximately $5x10^3$/ml murine Mlg fibroblasts were seeded in 96-well plates. Then 100 µl of culture supernatants from MLE12/pBI-L-CHOP-overexpressing MLE12 cells or MLE12/pBI-L-EV-expressing cells were transferred to Mlg cells and incubated for additional 24h. The absorbance of each well was then determined by a spectrophotometer (SpectraFluor Plus, Tecan) at either 450 nm for WST-1 assay or 370 nm for BrdU assay.

Cytotoxicity assay

The Cytotoxicity Detection Kit (Roche) contains a colorimetric assay for the quantification of cell death and cell lysis, based on the measurement of lactate dehydrogenase (LDH) activity released from the cytosol of damaged cells into the cell culture medium. LDH assay was performed according to manufacturer's instructions. Briefly, MLE12/pBI-L-CHOP or MLE12/pBI-L-EV cells were grown in 12-well plates in complete medium containing 100 µg/ml of G418 and hygromycin. Cells were treated for indicated time points with 1 µg/ml of doxycycline (Sigma) or left untreated. Cell supernatants were then mixed with the Reaction Mix from the kit, and the absorbance was measured at 490 nm by a spectrophotometer (SpectraFluor Plus, Tecan).

Immunohistochemistry

ZytoChem-Plus AP Kit (Fast Red) (Zytomed Systems, Berlin, Germany) was used for immunohistochemical localization of CD68, c-Ets-1, AP-1, proSP-C, HOPX and CHOP in formalin-fixed, paraffin-embedded lung tissue sections from patients with sporadic IPF (n=7) and organ donors (n=4), according to the manufacturer´s instructions and previous published work [S9]. Human lungs were placed in 4% (w/v) paraformaldehyde after explantation (fixation was done for 12 – 24h), and processed for paraffin embedding. Sections (3 µm) were cut and mounted on positively charged glass slides (Super Frost Plus, Langenbrinck). Paraffin-embedded tissue sections of normal donor and IPF lungs were deparaffinized in xylene and rehydrated in graded alcohol. Antigens were retrieved by cooking the sections for 5 min in 10 mmol/L citrate buffer (pH 6.0) using microwave irradiation (800 W). Thereafter, sections had to cool down for

20 min at RT, followed by repeated cookings (800 W, 5 min) and coolings (20 min at RT). This procedure was performed three times. Importantly, the citrate buffer was freshly prepared by mixing 18 mL 100mmol/L citric acid monohydrate and 82 mL 100mmol/L sodium citrate tribasic dihydrate with 900 mL distilled water.

For immunostaining, the streptavidin-biotin-alkaline phosphatase (AP) method with use of the ZytoChem-Plus AP Kit (Fast Red) [Zytomed Systems, Berlin, Germany], according to the manufacturer´s protocol, was employed. In the following, the primary antibodies used for IHC are listed, including the sources and dilutions: CD68 (1:200, abcam, ab955), c-Ets-1 (1:50, abcam, ab26096), AP-1 (1:50, abcam, ab31419), proSP-C (1:750, Millipore, AB3786), HOPX (1:50, Santa Cruz, sc-398703), CHOP (1:200, CST, #5554) and another CHOP antibody (1:50, Santa Cruz, sc-7351).

In general, sections were incubated for 2h at RT with primary antibodies, which were diluted in PBS containing 2% (w/v) BSA. Control sections were treated with PBS-2%BSA alone to determine the specificity of the staining. Detection was performed with a polyvalent secondary biotinylated antibody (rabbit, mouse, rat, guinea pig, provided by the ZytoChem-Plus AP Kit, 20 min incubation) followed by incubation with AP-conjugated streptavidin (20 min). Sections were then developed with Fast Red substrate solution (red stain), and the reaction was terminated by washing in distilled water. The stained sections were counterstained with hemalaun (Mayers hemalaun solution, WALDECK Division CHROMA GmbH & CO KG, Münster, Germany) (blue stain) and mounted in Glycergel (DakoCytomation). Lung tissue sections were scanned with a scanning device (Nano-Zoomer, Hamamatsu), and examined histopathologically using the ´NDP.view2 software´ at 100×, 200×, 400× and 800× original magnification.

Immunofluorescence

Human lung tissue was fixed in 4% paraformaldehyde for 12-24h, and processed for paraffin embedding. Paraffin-embedded lung tissue sections (3 μm) were deparaffinized in xylene and rehydrated in decreasing concentrations of alcohol. Antigens were retrieved by boiling the sections in the microwave for 20 min in 10 mM citrate buffer (pH 6.0). In the following, the primary antibodies used for immunofluorescence are listed, including the sources and dilutions: Anti-HTII-280 (1:300, Terrace Bioteck, #TB-27AHT2-280 Ms IgM), anti-CD68 (1:100, abcam, ab955), anti-c-Ets-1 (1:100, abcam, ab26096), anti-AP-1/c-Jun (1:300, abcam, ab31419) and anti-CHOP (1:100, CST, #5554). Sections were then incubated with the respective Alexa Flour 488, 555 or 647-labeled donkey anti-rabbit or -mouse IgG/IgM-antibodies (all Invitrogen). In detail, anti-rabbit IgG (H+L), F(ab')2 Fragment, Alexa Fluor® 555 Conjugate, #4413, was used as secondary antibody for Chop, c-Ets-1 and AP-1 in a dilution of 1:1000; anti-mouse IgG (H+L), F(ab')2 Fragment, Alexa Fluor® 647 Conjugate, #4410, was used as secondary antibody for CD68 in a dilution of 1:1000; and goat anti-mouse IgM, Alexa Fluor® 555 Conjugate, #A21042, was used as secondary antibody for HTII-280 in a dilution of 1:3000. DAPI-staining (Sigma, #D9542) was used as nuclear counterstain. As negative (background) control, the first antibody was omitted, and lung tissue slides were only incubated with different fluorochrome-conjugated secondary antibodies. Sections were mounted in Fluorescence Mounting medium (Dako, #S3023). Imaging was performed using Axio Observer.Z1 fluorescence microscope (Carl Zeiss MicroImaging, Germany). Immunofluorescence images were acquired and analyzed using Axio Observer ZEN software, version 1.0 (Carl Zeiss MicroImaging).

Supplementary References

[S1] R Development Core Team (2007) R: A language and environment for statistical computing. R foundation for Statistical Computing, Vienna, Austria. ISBN 3-900051-07-0. Available: http://www.R-project.org.

[S2] Ritchie ME, Phipson B, Wu D, Hu Y, Law CW, Shi W, Smyth GK (2015) limma powers differential expression analyses for RNA-sequencing and microarray studies. *Nucleic Acids Res* 43: e47

[S3] Gentleman RC, Carey VJ, Bates DM, Bolstad B, Dettling M, Dudoit S, Ellis B, Gautier L, Ge Y, Gentry J, Hornik K, Hothorn T, Huber W, Iacus S, Irizarry R, Leisch F, Li C, Maechler M, Rossini AJ, Sawitzki G, Smith C, Smyth G, Tierney L, Yang JY, Zhang J (2004) Bioconductor: open software development for computational biology and bioinformatics. *Genome Biol* 5: R80

[S4] Edwards D (2003) Non-linear normalization and background correction in one-channel cDNA microarray studies. *Bioinformatics* 19: 825-833

[S5] Mahavadi P, Korfei M, Henneke I, Liebisch G, Schmitz G, Gochuico BR, Markart P, Bellusci S, Seeger W, Ruppert C, Guenther A. (2010) Epithelial stress and apoptosis underlie hermansky-pudlak syndrome-associated interstitial pneumonia. *Am J Respir Crit Care Med* 182: 207-219

[S6] Rice WR, Conkright JJ, Na CL, Ikegami M, Shannon JM, Weaver TE (2002) Maintenance of the mouse type II cell phenotype in vitro. *Am J Physiol Lung Cell Mol Physiol* 283: L256-264.

[S7] Livak KJ, Schmittgen TD (2001) Analysis of relative gene expression data using real-time quantitative PCR and the 2(-Delta Delta C(T)) Method. *Methods* 25: 402-408

[S8] Acosta-Alvear D, Zhou Y, Blais A, Tsikitis M, Lents NH, Arias C, Lennon CJ, Kluger Y, Dynlacht BD (2007) XBP1 controls diverse cell type- and condition-specific transcriptional regulatory networks. *Mol Cell* 27: 53-66

[S9] Korfei M, Skwarna S, Henneke I, MacKenzie B, Klymenko O, Saito S, Ruppert C, von der Beck D, Mahavadi P, Klepetko W, Bellusci S, Crestani B, Pullamsetti SS, Fink L, Seeger W, Kramer OH, Guenther A (2015) Aberrant expression and activity of histone deacetylases in sporadic idiopathic pulmonary fibrosis. *Thorax* 70: 1022-1032.

Supplementary Tables S1-S6

Table S1: Primer sequences used for cloning of genes

Gene	Gene Bank number	Forward 5' - 3'	Reverse 5' - 3'
Chop	NM_007837	GATGCGGCCGCGCCACC**ATG**GCAGCTGAGTCCCT	GATGTCGACTCATGCTTGGTGCAGGCT
Chop	NM_007837	CTTGATGTCGACGCCACC**ATG**GCAGCTGAGTCCCTGC	GGTCATAAGCTTTCATGCTTGGTGCAGGCTGAC
Sp-1	NM_013672	CTTGATGAATTC**ATG**AGCGACCAAGATCACTC	GGTCATCTCGAGGAAACCATTGCCACTGATATTAAT
Ets-1	NM_011808	CTTGATGGATCC**ATG**AAGGCGGCCGTCGATC	GGTCATGTCGACGTCAGCATCCGGCTTTACAT
Jun	NM_010591	CTTGATGGATCC**ATG**ACTGCAAAGATGGAAACGA	GGTCATCTCGAGAAACGTTTGCAACTGCTGCG
Mzf1	NM_145819	CTTGATGAATTC**ATG**AGACCCACTGTGCTGGGCTCC	GGTCATCTCGAGCTCAGTGCTGTGGACACGCTGGT
Atf4	NM_009716	GATGCTAGCGCCACC**ATG**ACCGAGATGAGCTTC	GATCCCGGGCTATTACGGAACTCTCTTCTG
Atf6	NM_001081304	GATGCTAGCGCCACC**ATG**GAGTCGCCTTTTAG	GATCCCGGGCTAACAGACAGCTCTTCGCTTTGG
Xbp1	NM_001271730	GATGCTAGCGCCACC**ATG**GTGGTGGTG	GATCCCGGGTTAGACACTAATCAGCTGGGG

Restriction enzyme sites are underlined. GCCACC = Kozak-sequence.

Table S2: Primer sequences used for cloning of human gene promoter fragments

Fragment name	Forward 5' - 3'	Reverse 5' - 3'
1st fragment (*CHOP*)	CTTGATGGTACCCGGCTAATTTTTGTATTTTTAGTA	GGTCATAAGCTTGACCTCGGGAGCGCCTG
2nd fragment (*CHOP*)	CTTGATGGTACCTCCCCTGCGCGTGCGCG	GGTCATAAGCTTGCCGACCTCGGGAGCTG
3rd fragment (*CHOP*)	CTTGATGGTACCCGGCTAATTTTTGTATTTTTAGTA	GGTCATAAGCTTCACCGAGGGTGGTGGGAG
4th fragment (*CHOP*)	CTTGATGGTACCTCCCCGCCCCCCTTTCCT	GGTCATAAGCTTGCCCCGCCCCGTGCCT
5th fragment (*CHOP*)	CTTGATGGTACCCAAGTCACATGACCTCTGCC	GGTCATAAGCTTGGTGTGCTGATGCGCGCCT
ACTB promoter	CTTGATGGTACCGAAAGGGTGACAAGGACAGG	GGTCATAAGCTTTACCCCCTCTCCCCTCCTT

Restriction enzyme sites are underlined.

Table S3: Primer sequences used for cloning of small DNA fragments of the 4th fragment of the human *CHOP* promoter

Fragment name	Forward 5' - 3'	Reverse 5' - 3'
4.1 fragment	CTTGATGGTACCCCTTTCCTCCCCTCCCCC	GGTCATAAGCTTGCCCCGCCCCGTGCCT
4.2 fragment	CTTGATGGTACCCCCGCTACACTCCCCTC	GGTCATAAGCTTGCCCCGCCCCGTGCCT
4.3 fragment	CTTGATGGTACCGCGCGCATGACTCACCCA	GGTCATAAGCTTGCCCCGCCCCGTGCCT
4.4 fragment	CTTGATGGTACCCCTCCTCCGTGAAGCCTC	GGTCATAAGCTTGCCCCGCCCCGTGCCT
4.5 fragment	CTTGATGGTACCTCCGACACTACGTCGACCC	GGTCATAAGCTTGCCCCGCCCCGTGCCT
4.6 fragment	CTTGATGGTACCTCCCCGCCCCCCTTTCCT	GGTCATAAGCTTGTCCCTCGCATCCGCCA
4.7 fragment	CTTGATGGTACCTCCCCGCCCCCCTTTCCT	GGTCATAAGCTTGGCTTTGGGTCACGAGGC
4.8 fragment	CTTGATGGTACCTCCCCGCCCCCCTTTCCT	GGTCATAAGCTTGCGCGCCGCGGAGGG
4.9 fragment	CTTGATGGTACCTCCCCGCCCCCCTTTCCT	GGTCATAAGCTTGGAGTGTAGCGGGGGGG
4.10 fragment	CTTGATGGTACCTCCCCGCCCCCCTTTCCTCC	GGTCATAAGCTTAGGGGCGGGGGGAAAGGAGG

Restriction enzyme sites are underlined.

Table S4: Primer sequences used for site-directed mutagenesis of the AP-1 and c-Ets-1 DNA binding-sites in the 4th fragment of the human *CHOP* promoter

Fragment name	Forward 5' - 3'	Reverse 5' - 3'
CHOP-AP-1	CGCGGCGCGCAgGACTCACCCAC	GTGGGTGAGTCcTGCGCGCCGCG
CHOP-c-Ets-1	CAAAGCCACTTCtGGGTCCGACACT	AGTGTCGGACCCaGAAGTGGCTTTG

The sites of point mutations for AP-1 and c-Ets-1 DNA binding-sites are indicated in lowercase letters.

Table S5: Primer sequences used for quantitative (q)PCR

Gene	Gene Bank number	Forward 5' - 3'	Reverse 5' - 3'
Actb	NM_007393	CTACAGCTTCACCACCACAG	CTCGTTGCCAATAGTGATGAC
Atf3	NM_007498	ACAACAGACCCCTGGAGATG	CCTTCAGCTCAGCATTCACA
Atf4	NM_009716	TTTGACAGCTAAAGTGAAGACTGA	GCTTCTTCTGGCGGTACCTA
Atf6	NM_001081304	AATGCCAGTGTCCCAGCAA	GCGCAGGCTGTATGCTGA
Atg12	NM_026217	AACAAAGAAATGGGCTGTGG	GAAGGGGCAAAGGACTGATT
Bad	NM_007522	CCACCAACAGTCATCATGGA	AACTCATCGCTCATCCTTCG
Grp78	NM_022310	TGGGAGGAGTCATGACAAAA	GGGGTCGTTCACCTTCATAG
B2m	NM_009735	GCTATCCAGAAAACCCCTCAA	CATGTCTCGATCCCAGTAGACGT
Chop (Ddit3)	NM_007837	CCTAGCTTGGCTGACAGAG	GTCAGGCGGTCGATTTCC
Edem	NM_138677	TGGAATTTGGGATTCTGAGC	CATAGAAGGAATCCAGCCCA
Erdj5	NM_024181	AGCTGGGGTCTTGGATTTTT	CGGCTCTCACTTTTCCTTTG
Ets1	NM_011808.2	CCGCCAAGTGCCAACTTC	AGCTTTCAAGGCTTGGGACA
Grp94	NM_011631	CAGAGACTGTTGAGGAGCCC	CTTTGGATGGTCTCTGCCAT
Hmbs	NM_013551	CAAGAGTATTCGGGGAAACCTCAA	CAGGATCGTGCAACACACTCACTA
Jun	NM_010591.2	GGGAGCATTTGGAGAGTCCC	TTTGCAAAAGTTCGCTCCCG
Nfya	NM_001110832	AGTCAGTGGAGGCCAGCTTA	CCAGGAGGCACCAACTGTAT
Nrf2	NM_010902	GACTCGGTCCAGCGCAGTCG	TTCAGGCTCGGGGGCAGGTT
Ppib	NM_011149	TTTTGCTGCCCGGACCCTCC	TCCTGTGCCATCTCCCCTGGT
Spliced *Xbp1*	NM_001271730	GCTTTTACGGGAGATAACTC	GCCTGAACCTGCTGCG
Vcp	NM_009503	AGGTGCCACAAGTAACCTGG	GCCAGTAAGGTTTTCCCACA
ACTB	NM_001101	ACCCTGAAGTACCCCATCG	CAGCCTGGATAGCAACGT
CHOP (DDIT3)	NM_004083	ACTCTCCAGATTCCAGTCAGAG	GCCTCTACTTCCCTGGTCAG
ETS1	NM_005238	CAGCCTGAAAGGTGTAGACTT	TTCCGAGCTGATGGGATGG
JUN	NM_002228	GCAAACCTCAGGAACTTCAAC	TTCTCTCCAGCTTCCTTTTTC
SFTPC	NM_003018	CTCATCGTCGTGGTGATTGTG	CTGCAGAGAGCATTCCATCTG

Table S6: Results of gene set enrichment analysis

	Treatment / transfection time		22h			44h			66h			44h	0,5h	1h	6h
	Regulation by		Atf4	Atf6	Xbp1	Atf4	Atf6	Xbp1	Atf4	Atf6	Xbp1	TG	TG		
No.	Name of pathway	No. of genes													
4110	Cell cycle	111	y	y	y	y	y	y		y	y	y			y
4120	Ubiquitin mediated proteolysis	131	y	y	y	y	y	y		y	y	y			
5220	Chronic myeloid leukemia	75	y	y	y	y		y	y	y	y	y			
4010	MAPK signaling pathway	258	y	y	y	y			y	y	y	y			
4012	ErbB signaling pathway	86	y	y	y	y			y	y	y	y			
4360	Axon guidance	127	y	y	y	y			y	y	y	y			
4510	Focal adhesion	190	y	y	y	y		y		y	y	y			
4810	Regulation of actin cytoskeleton	201	y	y	y	y		y		y	y	y			
5215	Prostate cancer	90	y	y	y	y		y		y	y	y			
5222	Small cell lung cancer	85	y	y	y	y	y			y	y	y			
510	N-Glycan biosynthesis	42		y	y	y	y	y			y	y			
4150	mTOR signaling pathway	51	y	y	y	y				y	y	y			
4540	Gap junction	87	y	y	y	y				y	y	y			
4910	Insulin signaling pathway	133	y	y	y	y				y	y	y			
5211	Renal cell carcinoma	70	y	y	y	y				y	y	y			
5212	Pancreatic cancer	72	y	y	y			y		y	y	y			
5213	Endometrial cancer	52	y	y	y	y				y	y	y			
5214	Glioma	65	y	y	y	y				y	y	y			
5219	Bladder cancer	41	y	y	y	y				y	y	y			
5221	Acute myeloid leukemia	56	y	y	y	y				y	y	y			
5223	Non-small cell lung cancer	54	y	y	y	y				y	y	y			
100	Biosynthesis of steroids	23	y	y		y	y				y	y			
4130	SNARE interactions in vesicular transport	34	y	y	y	y	y				y				
4210	Apoptosis	85	y	y	y					y	y	y			
4512	ECM-receptor interaction	77	y	y			y			y	y	y			
4520	Adherens junction	74	y	y	y					y	y	y			
4530	Tight junction	125	y	y	y					y	y	y			
4070	Phosphatidylinositol signaling system	69	y	y	y					y	y				
4115	p53 signaling pathway	66	y	y	y					y		y			y
4370	VEGF signaling pathway	73	y	y	y						y	y			
4920	Adipocytokine signaling pathway	64	y	y	y					y	y				
5210	Colorectal cancer	85	y	y	y	y						y			

Table S6: Results of gene set enrichment analysis (continued)

	Treatment / transfection time		22h			44h			66h			44h	0,5h	1h	6h
	Regulation by		Atf4	Atf6	Xbp1	Atf4	Atf6	Xbp1	Atf4	Atf6	Xbp1	TG	TG		
No.	Name of pathway	No. of genes													
5218	Melanoma	70	y	y						y	y	y			
20	Citrate cycle (TCA cycle)	29	y	y						y	y				y
290	Valine, leucine and isoleucine biosynthesis	11		y		y				y	y				
562	Inositol phosphate metabolism	47	y	y	y					y					
680	Methane metabolism	8		y	y	y						y			
1030	Glycan structures- biosynthesis 1	113		y			y				y	y			
3022	Basal transcription factors	31		y	y					y		y			
4310	Wnt signaling pathway	148	y	y	y							y			
4340	Hedgehog signaling pathway	51	y	y	y							y			
4350	TGF-beta signaling pathway	86	y	y	y							y			
4912	GnRH signaling pathway	95	y	y		y						y			
5040	Huntington's disease	31	y	y	y						y				
5050	Dentatorubropallidoluysian atrophy (DRPLA)	15	y	y	y						y				
51	Fructose and mannose metabolism	37								y	y	y			
230	Purine metabolism	148		y					y		y				
240	Pyrimidine metabolism	91	y	y								y			y
563	Glycosylphosphatidylinositol (GPI)-anchor biosynthesis	22	y	y	y										
970	Aminoacyl-tRNA biosynthesis	42		y		y						y			
1031	Glycan structures- biosynthesis 2	59		y	y						y				
3410	Base excision repair	33		y	y						y				
4140	Regulation of autophagy	29	y	y	y										
4620	Toll-like receptor signaling pathway	94	y	y	y										
4650	Natural killer cell mediated cytotoxicity	117	y	y			y								
4660	T cell receptor signaling pathway	96	y	y	y										
4662	B cell receptor signaling pathway	67	y	y	y										
4664	Fc epsilon RI signaling pathway	78	y	y								y			
4720	Long-term potentiation	67		y						y		y			
4930	Type II diabetes mellitus	43		y	y				y						

Table S6: Results of gene set enrichment analysis (continued)

	Treatment / transfection time		22h			44h			66h			44h	0,5h	1h	6h
	Regulation by		Atf4	Atf6	Xbp1	Atf4	Atf6	Xbp1	Atf4	Atf6	Xbp1	TG	TG		
No.	Name of pathway	No. of Genes													
5216	Thyroid cancer	27	y	y	y										
52	Galactose metabolism	24								y	y				
251	Glutamate metabolism	24		y								y			
310	Lysine degradation	44		y	y										
900	Terpenoid biosynthesis	5		y			y								
3020	RNA polymerase	27		y							y				y
3420	Nucleotide excision repair	41		y							y				y
4630	Jak-STAT signaling pathway	144	y		y										
4670	Leukocyte transendothelial migration	113		y								y			
5010	Alzheimer's disease	156		y								y			y
5217	Basal cell carcinoma	54		y								y			
71	Fatty acid metabolism	43								y					
120	Bile acid biosynthesis	28								y					
190	Oxidative phosphorylation	112										y			y
252	Alanine and aspartate metabolism	29										y			
280	Valine, leucine and isoleucine degradation	44										y			y
360	Phenylalanine metabolism	20				y									
400	Phenylalanine, tyrosine and tryptophan biosynthesis	9		y											
450	Selenoamino acid metabolism	22		y											
480	Glutathione metabolism	50										y			y
511	N-Glycan degradation	14									y				
520	Nucleotide sugars metabolism	6			y										
530	Aminosugars metabolism	30										y			
532	Chondroitin sulfate biosynthesis	21		y											
533	Keratan sulfate biosynthesis	16										y			
565	Ether lipid metabolism	31										y			
601	Glycosphingolipid biosynthesis - lacto and neolacto series	24									y				
604	Glycosphingolipid biosynthesis - ganglio series	15										y			

Table S6: Results of gene set enrichment analysis (continued)

No.	Name of pathway	No. of Genes	22h			44h			66h			44h	0,5h	1h	6h
	Regulation by		Atf4	Atf6	Xbp1	Atf4	Atf6	Xbp1	Atf4	Atf6	Xbp1	TG	TG		
670	One carbon pool by folate	15										y			
750	Vitamin B6 metabolism	5								y					
760	Nicotinate and nicotinamide metabolism	23	y												
790	Folate biosynthesis	29		y											
791	Atrazine degradation	4		y											
920	Sulfur metabolism	11									y				
980	Metabolism of xenobiotics by cytochrome P450	65										y			
982	Drug metabolism - cytochrome P450	74										y			
1032	Glycan structures - degradation	26				y									
1040	Biosynthesis of unsaturated fatty acids	30									y				
3030	DNA replication	35		y											y
3060	Protein export	11		y											
3440	Homologous recombination	27		y											y
4330	Notch signaling pathway	47									y				
4710	Circadian rhythm	13		y											
4916	Melanogenesis	100										y			
5012	Parkinson's disease	110										y			y
5014	Amyotrophic lateral sclerosis (ALS)	58		y											
5340	Primary immunodeficiency	36			y										
624	1- and 2-Methylnaphthalene degradation	9										y			

(Column header spanning the time row: Treatment / transfection time)

The table lists the KEGG-pathways significantly regulated (adj. $p < 0.05$) in at least one of the contrasts from the arrays. A “y” labels the individual contrasts in which the pathway was regulated. TG 0.5 h, TG 1 h and TG 6 h stand for the contrasts of TG-treated versus WT-MLE12-cells. The pathway analysis for those contrasts was performed separately. Abbreviations: TG = thapsigargin.
