# Supplementary material for: Regulation and role of the ER stress transcription factor CHOP in alveolar epithelial type-II cells
Source: J Mol Med (Berl). 2019 Apr 26;97(7):973–90. doi: 10.1007/s00109-019-01787-9 (PMC6581940; doi:10.1007/s00109-019-01787-9)
Supplement: Supplementary file 2 — (PDF 6820 kb) [file 109_2019_1787_MOESM2_ESM.pdf]

Regulation and role of the ER-stress transcription factor CHOP in alveolar epithelial type-II cells

Supplementary Figures S1-S12

Oleksiy Klymenko[1,2], Martin Huehn[1,2], Jochen Wilhelm[1,2], Roxana Wasnick[1,2], Irina Shalashova[1,2], Clemens Ruppert[1,2,3], Ingrid Henneke[1,2], Stefanie Hezel[1,2], Katharina Guenther[1,2], Poornima Mahavadi[1,2], Christos Samakovlis[1,2,3,4], Werner Seeger[1,2,3,5], Andreas Guenther[1,2,3,6,7]*‡, Martina Korfei[1,2‡].

‡These authors contributed equally to this work.

Affiliations:

[1]Department of Internal Medicine, Justus-Liebig-University Giessen, D-35392 Giessen, Germany.

[2]Universities of Giessen and Marburg Lung Center (UGMLC), Member of the German Center for Lung Research (DZL), D-35392 Giessen, Germany.

[3]Excellence Cluster Cardiopulmonary System (ECCPS), D-35392 Giessen, Germany.

[4]Department of Molecular Biosciences, The Wenner-Gren Institute, Stockholm University, SE-106 91 Stockholm, Sweden.

[5]Max-Planck-Institute for Heart and Lung Research, Department of Lung Development and Remodeling, D-61231 Bad Nauheim, Germany.

[6]European IPF Network and European IPF Registry.

[7]Agaplesion Lung Clinic Waldhof-Elgershausen, D-35753 Greifenstein, Germany.

***Corresponding Author**
Andreas Guenther, M.D.
Department of Internal Medicine
Justus-Liebig-University Giessen
Klinikstrasse 36
D-35392 Giessen, Germany
E-mail: Andreas.Guenther@innere.med.uni-giessen.de
Tel.: +49 641 98542502
Fax: +49 641 98542508

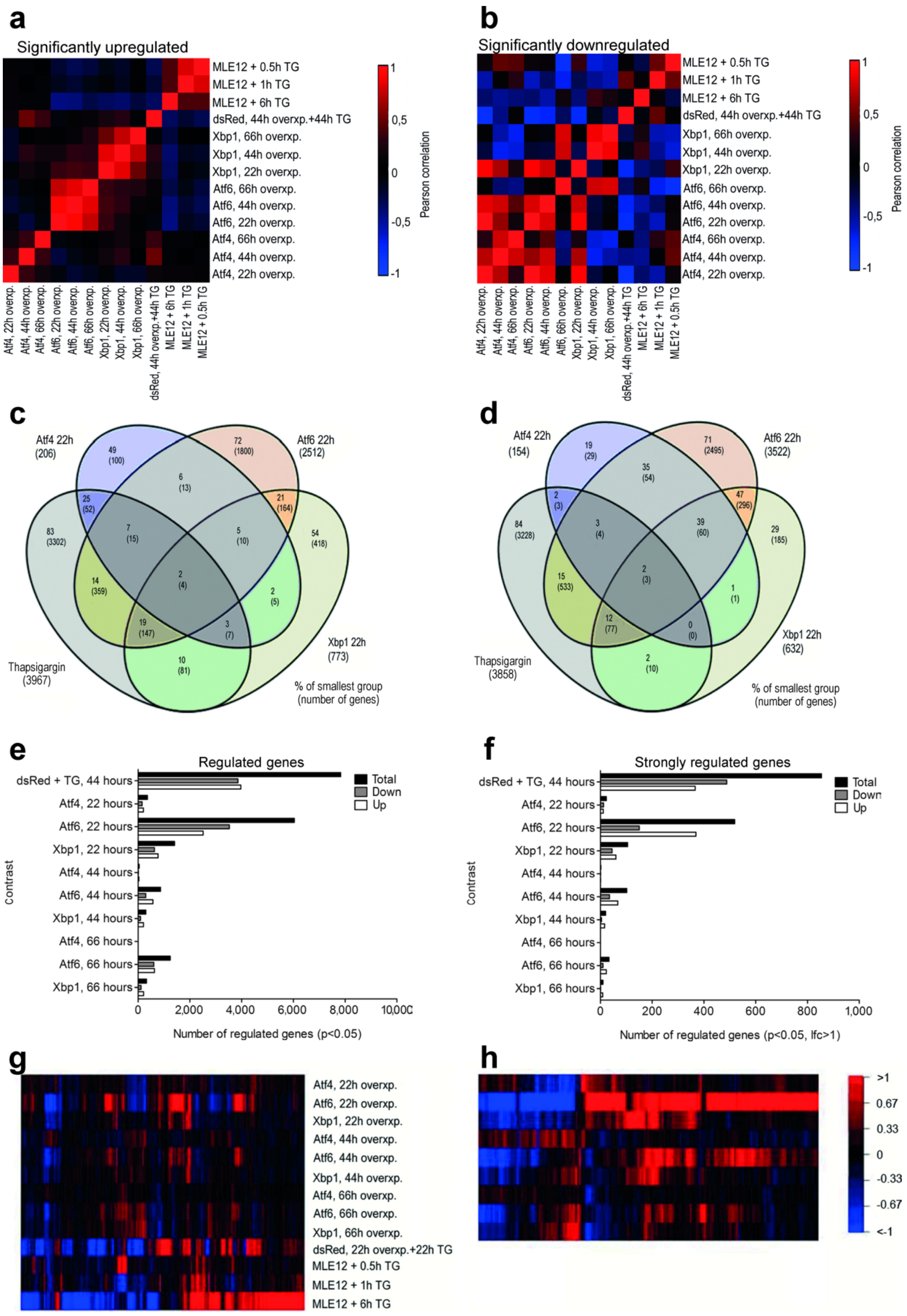

Fig. S1. Correlation of the effects elicited by overexpression of Atf4, p50Atf6 and sXbp1 and thapsigargin-effects.

(**a**, **b**) Color-coded correlation charts of UPR transcription factor effects and thapsigargin (TG)-effects: Whole genome expression analysis using RNA from MLE12 cells overexpressing Atf4, p50Atf6 or sXbp1, versus empty-vector (dsRed) and TG (1 µM/ml)-treated cells (n=6 experiments per condition for the time periods 22h, 44h, 66h; and n=4 for the 0.5h, 1h and 6h treatments with TG, and n=12 for the respective control). Only significantly up-regulated genes are included (based on a 5% false-discovery rate). The color coding (red = Pearson correlation coefficient of 1; blue = Pearson correlation coefficient of -1) indicates the extent of correlation between the different conditions. (**b**) Same as in (**a**), but only significantly down-regulated genes are included.

(**c**, **d**) Venn-diagrams, depicting the number of genes up-regulated (**c**) or downregulated (**d**) based on a 5% false-discovery rate and their relative distribution in subgroup and conditions.

(**e**, **f**) Extend of the transcriptomic response of MLE12 cells to single overexpression of Atf4, p50Atf6, sXbp1 or treatment with TG. Given is the number of regulated genes with $p < 0.05$ in (**e**) or strongly regulated genes ($p < 0.05$, $lfc > 1$) in (**f**).

(**g**, **h**) Time-dependent changes in transcription regulation in response to Atf4, p50Atf6 and sXbp1 overexpression and TG effects in MLE12 cells. The left heat map (**g**) includes the effects of TG and the effects of the transgene expression (selection for both: genes with $p < 0.05$ and $lfc>1$ in at least one contrast). On the right one (**h**), exclusively the effects of single transgene expression are depicted [on the same lane as in (**g**)]. Red and blue indicate increased and decreased gene expression, respectively.

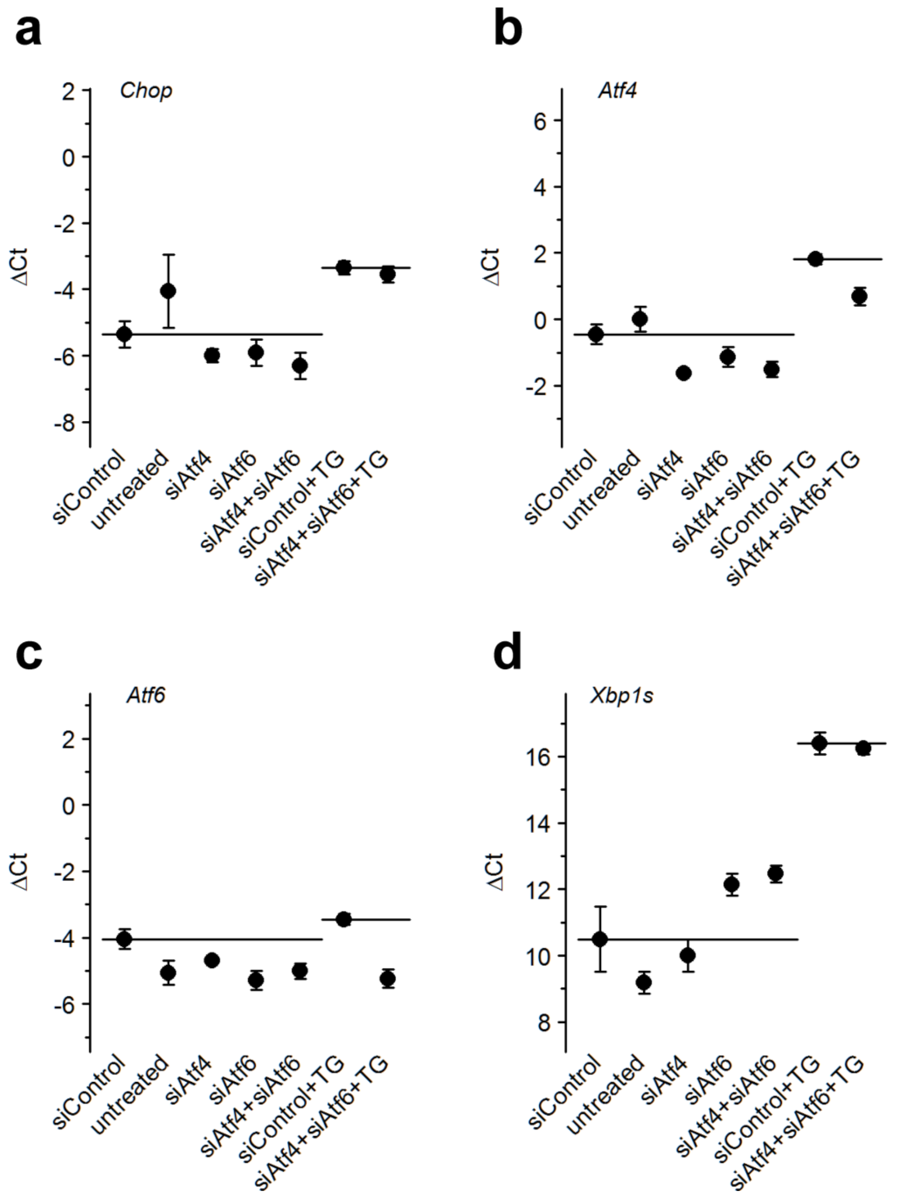

Fig. S2. Effects of Atf4 and/or Atf6 knockdown on thapsigargin (TG)-induced and non-induced *Chop-expression* in MLE12 cells.

(**a**-**d**) Quantitative RT-PCR analysis for *Chop* (**a**), *Atf4* (**b**), *Atf6* (**c**) and spliced *Xbp1* (*Xbp1*s) (**d**) in untreated MLE12 cells, and cells transfected with non-targeting siRNA (siControl), *Atf4* and/or *Atf6* targeting siRNA in the presence (1 μM/ml) or absence of TG. Data are expressed as means ± SD, from n=4 independent experiments.

CHOP Promoter
>*DDIT3* 9999bp genomic DNA (5´- 3´)

GCTAGGGGTTGGTTATCATCAGAAGGTGGTCTCCCCTCAGGCCTGGGGGATAAGGACGTGGGCCCAGC
CACATGCCAACTCATGTCCAATACTGCTTTGCCTGGTGTGGGGAAGGATTGGGTCTTGTCCCCCAACAC
AGCTTCTGTGGCTGACTGTAATACTGTACAACTGTTTCTGACCATTAAATGCTGTTGTACTCTGTGTGG
CCTCTGCTGTGTTTCCTGGGGAGGAAGCAGCACTAGGATATAGATATTCATTCGTCATAACAGGCAAT
CTAAGCCACTCTATACTACAAGAGATGGATTTAAATTGTAACCTGTTCTTACCAAAGAACTAAATAAA
AAATGAGTACAGAGCCAGAGCCAGAGTTTCAAAATATTCTCATCTGTTAAATTAAGAGTGTCTCCCAT
AGAAAAGCAGTGGAGGCCCCACAGGGCAAGTACAAAACAGAATTAAAACTCCCAAGGGTCTTGTCTT
TACAAAAGAAAAGGCAGGAGGCAGCCCCTGGACAGCTGGTCATGCTGGCCGCTCCGGTTGGACCACG
TTGCATAATCCTCAGTCGCATCATCACAACGTCTCTGAGCGTTTTGATGGGGGGAGAAGGGGCAGTGT
AGTGTGTATGGGAGGAGAGGCCCAGAGGGCTCTCTTTGCCCCCTTACCCCCTTTTTTATATCCCAGAGG
AAAGTCGGGGGAACCTGGCTACACCTTGAAATGAGGCTATGTGTTTCAAACCTGGGGACGGGGTAAG
AGAGGATCTGTGCTTTGAGCAACCTGAGCCAGAGGCAGAGGGGTGTTGGAGGGGTAAGGGGAGGATG
CATGATGCTTATTGCTTTGTACCTTTCACTGGGAAGGAGGGCAGCAGCCAACAGTAGCTCACAGGTTT
GTAAACTGAGCCTGTTGGCTTTAAGAAGGGAGGCAATGAAATCGAATTAAATATAAAAGAGTCATTTG
TGCAAAAATAACTTAAACAAATAAAAGACCTGGGGAAGGGGGTGTTCCCCTTAGCGCCTGGTGGGGA
AAGGGCCATATACCATCCCCCCCAGGCCTTTTCAGTGACATGGCTTCGGGGGGGCGGGGGGGTGGTGG
GGGGGGGTGAACTTCCCTGCCCCCTGCAATGGCTCAGGATGGGATTGTAGGGGAAGGAGTTGCATTTG
TGCTCTGAGTGGGGAGTAGTGCCCCCACCCACTGTCCACAGGTGCAGGTGGCTGGCAGGGGCTCCCAA
GGCTCAGCACTCAGCTCTCCCCAATCAGGGTCAGATCCAGCTCCAGGTATGGCTGCTATGGGGCCAGT
TTCCTCCTCTTGTTTTTGGCAGGACGGCCAGGGCGGGCCCGGGGAGGCAGAGGGACAGCTGCCTAATA
TCAGAGGGGAAGTTGATAGAAGTGGTCAATTGCTAGGTATCAGGAAGTCTTCTTTTCTCCCCACTCCCT
CTAACCCTCCTTTCCTCCTGCCGCCTCATCCCTTACACCCTACCCATCCACCCATCCACCCAAACACAC
ACTTACTCGGGCTGTAGGGCTGGGTTCCAAGGTAATGTCCTGGCGGGAGCTATTGCTGTTCCGGGTAG
GGCTGCAATAAGGGGCACAGATGATCAGGAACAGCTCCCAAGCACCAGGTGAATTTCAGAAGTTTGG
GTGGGAAACTTTCTACATTTGGATAGTAAAGGCATAATATCCTTATTCATATTCAAATAAGAATCTGCC
CTTTTGAAAACCAAGCCACTATTTGGACATTAGAACTGTCCAAACCAAAAAGAAGTAGGCAAAGGCC
AGCACTATAGTAGGCCCAACACTCACTTGTATTTTCTCCTACGGCCACGACGAGACTTTCTGACTACTC
CCGGGGGCACCTGAGAAAAGATGGACTATGTGTGTGGGAAATTTCGAAAAAGGCTCCAAAGCCCCAA
CCTTTCCTCAGAGCCCCTGAGCCCAGACTACCAGCACTCTGCACTCACCTTAAGGTCCTCAGAATGGG
GCCCGGGAGGGGGTGGATCCTTGGGCTCAGCACCCCCTTCAGCCAGCTCCCCATTATGCTGCCAATGG
TGGGTTCTTCTTGGGGACCTTTCCATTTGTCCATTGAAGCCACCACGTGTCCCCCATTTGAGGGCCAGT
CGGCCAGGCTCCCGCCGGCTGCCAGGCTTTCGGCCCCGGGCTGGCCTGGCCTCACCATTAATGCCCCT
AAGTCCCCCTCCTCCCCTCCGGCCCCGTTTCCCTGACCCCCTCCCAGTGAGCAGCTCAGGGACTGGGGG
GTCGGGAGAACCATGGGGTGGGGCTAAGGCACTGAGGGGAGGCCTGGCAGGCTCTGGTTCGAGGGCT
GAGGCAGGGCTCTCGGGGGGTAGACAGGGGGCTTCTGGCTGCTCTGGAGGGATCTGCTGGCAAAAAA
ATAAGTTCTCAGTCAATCTCAAATACCGTCCCTCTTCTACTATAACTCAGAAAGCCCTAAATTTCCAGT
TTGAGAAAAAATTATCTCCCTGACAAATATATGCCCACACCCTCACCTGGAGACTCTGTAGCAGGCAG
GCTAGGGGACTAGAAGCCTCTGAGAGGGGTGGAGGAGCTCCCCCCCCAGGGAGGAGCTGCAGCCCCA
ACTGGCCAGAGAGAAGAGGGCCAGGAGGCTGCAACAGGCTGGGGAGGGCTCCAGGGCTGCTGGTCAG
TGAAGACAGGTCACCTGTTGAGAAATTATAGTCAGGGGTCTCTCCCTAGGCAAACCAATTAGGCTGAG
CAGACCTACTATTCTCTTTAGGCTTTATCCAGTGGCCAAAGACTTTCTTCCCCAATTTATTCAGCCACTA
CTCCCAAGTTTACTCCTGCTTTTTCACACCAAGAGTGTGAATTCACACTCCCTCAGACTCACCCAGCA
GGCTGGCACCCAGCAGAGGGCTAAGGAGTTGGGGGGGTCTCCCTGAGTTGGAGGGGGCCTTGCCCAG
AGAGGAGGCCCCCGGGTCAGTAGTTGCCGTGGTGACACTGGAGGTAGGTGGTCCAGGTTGGGGGGCA
CTCAGGACACAGGGCTAAGAAAAAAGAATGATTCTAACACTTACGCGTGTAAAATACTCAACAGATTT
ATAGTGCTTTCACATCCACTATCTGACTGAAGCCTCAAGTGAGATAGGCCATGCTTATTAAATACAGA
CCCAATTCTTGCAGTCATCCTACCCCCAGCCAAGAACTAAGGCCTCCCCAAAGGCCCCAAGGAAGAGC
TCAGGAATGAAACTGAAAGCTTTTGATTCCCAGCTCCCATCTCTTATTCTACATCTTGTTCTGTCCCACC
TACTCACCCCCATCCTCACCTGGGGGGGTGTCCCCGAGGGGGGATCCAGGGTGGCAGCCAGCAGCGCA

GAATTTAAAGCTATGAGGGTAGGGGGGGCTGAAAGTGGGGGAAAAGTAGGGGGGACAGGTCTCCCA
AGCCTGAAAATGGCTCCCCACTTGGACCCCCGGCTCCCTCTGCAGATCCCTCCCCATCCCCAGCTGTGG
GGCCCAGAGCCAACAGGGGCAGGAAAGAGGCAAGGAGGTTGCTGGGAGGTGCTGAAGGAGGTGGAA
GAAGGTCTGAGGGTGGTGGAGGAAGCAAGGAAGCCACCAAAAGCGAAGGCCCTTCAGGCTCTCCTGG
GGCTAGGGGAGGACCAGGTCCTCCTGGTGGGGGTAGCAGGGGCTCAGGGGGAGGTTGTCCTCCTCCCC
CAGTCAGCACACCAAATAAACTGTGGTTGAGCAATGGAGAAGGTGGAGGCTGCCCCAGACTCAGAGG
GAGAGGCAAGGCCCCAAGCATCCCAGGGAAGCCAGCTCCACTCAGTGCCAGGCCCTGCTCAGGGCTG
GGGAAAGGGAAAGCCTCTCCACCAGCCAGGGGAGGCAGAGGGCAGGCCGGGCCTGCCCCCATCCCCA
GTCCTTCGGATGGGCTTTGAAGCACAGGGCTGGGGACTACTGGGGCTTTGGAGGCAGCTGGTGGGGCA
GGGGCACCTGTAAAAGAGGAGAGAAATTCAGCTCAAGGGAGGCCCTTTCTCCCATCATCCAAAGAAG
CCAACCTCCCTGCCCCCAACCCAGAAAACCCTCAAAGAGCTCTCTGAACATTCCCCTAAATGCCTTGG
CTGCCTCTTTCCAGCCAGGATGAGGAAGGGCTCCACAATACTCACATACCTGGCACACTGCTGAGGCT
GCCACTGGTGGTCCCAGGCAGGGAGGGCTGGATGGGGTGCCTGGGCTGGGGAGGGCTCCCTGAGGAG
AGGGTTGGGGGAGGAGGAAGGTGTGCATCTGACCCCAAAAGGTTAAAGCTTCCATCAGAGTGGGAAG
GGCCAGGGGTGGGGAGTCCCAGCAGGGACAGCACAGAAGGGAGAATTGGTTGGGATGGCTCCGGGA
GAGAAAAGGGTTGGGGGACAGGAGCAGGGGCCCGAGGACGGCTCCGTCTAGGGGTTTGAGGGCCTCT
CCCTTCTAGCAATCGAAATACAGTAGGGGGTCTGCGGGGACGACGCTGAGAGGGACGAAGTGATGAG
GAGTGGGAAGCTGAGGGTGCCTGGGCACGGGGCCTTCGGCCCTGTAAAGTGCTGGGAGGGGGTAGTG
GGGGATGCTGTGCCTTGGCCGCTGCAGAGAGTAGGCTGCTAGCAAGGAAGGGGGGTGCCCCAGGCCC
CTCCACCGTGGGGGCCCCTCCCAGGGGCCCCAGGACCAGGGGCAGGTGCATAGTGGCTGAAGACACT
GGTGGCTGAGAGGCAGGACCAGGGTGGGCGGGGAGATTATTGCTCGGGGGCAGGGGAGGGGGTGTTA
AGGCATCACTACAGTGGAAGAGAGGAGGGTCTGAAGGTGGTGAGGAGGAGGCATGAGGGGCCGGAG
GAGAGCCCAGGTCAGAGGGCACCAGGCTGGATCTGAGGGCAGCTCCCCAGTTGTATGAGGGAGCATT
GAGGCTGATAGCAGGTGGAGGAGGTGGGGCTGGAGAGGGGGCATTGCCCCTTGGGAGGAAACGGGG
GCTGCTACTGCCCCCAGAAGGGACTGGGTCAGCAAGCCTTGGGGGGAAAAGCCCCCCAGGGCCTGCT
AGAGTGGGAAAGGCCTGGGAAACTGAGGGTGGTTCTGGGGGAAGGGAGCCAGGACCCCCATTAAGTG
GAGTTGTAGGAGGAACTCGACAGGGAGGGCGGGCAGAGGGGGGCCCTGGGGACACAGTGTGGAACA
TTTGGGGGCTCGCTCCCTCTCCTGCAATAAATGAAAAATGAAGGGTCAGCCTGTGGCTCCCCTTCCAA
AAACTCTTTTGTCTTCTCTAGTCCCATCTCCCACAAGTTCCCAACCTCCCTGTGCCCTCCTTTCAACATG
CAGCTCCCCACCCACTCCCCTTTCTCTTTGCTCCCTCTCAAGGAACTCCTCCCACCACCAGCCTTCCAAG
CCCTTAGGTTTTCCTCGGTCTTCTGTAAAGTGGCAGAAGACTCACCAGGAGAAGAGTGTGAGCAGGTG
GTCTCCATGCTGCGGTACAGAGTTGCCATAGCAACAGCTTTCCGCCGGTGGTTGCACAGCTTGGTCATG
TCCTCCTCTGATGCTGGCCCCACCCCAGCCCCACCCGGGGTCACCGGGGCCAAAGGGTCAAAGTTGAA
AACCTGTAAGGTGGAAAGCAAGGACAGGGACCCTGAGGGGCTGGAACCTGTGAGAAGGGCTACCTCA
GTCAGTGATTTGCATTAAGACTTTTAGCTTATCTCTGTACTCTCAGGCGCCCCTCACCACTCTGACCTTG
GGGACATTAAGTGGACACTCCAGACCGCACTTGCAGGTCCCATCGCTGAGGAGGTAGCTCCGGGTTTG
CTCCAAGGAAGACAGCTCTGTGCCACTTGGACTGCGAGAGGACAGGGTCAGAGGACATGGGGTTAGC
TGGCAGCGAATCAGGCAATACTCCTAAAGCACAGTGCTTAAGTTACAGATCCTCCTAGAAGAACAGAG
GATAAGGAATGTGGGAAGACACAGAAAAAGGGAGAATGGATTCAAAGGCCCAGAGCTACAGAAAGC
AAAAAGGCTGAGGAAGGAAGGGGAAGAGAGGAGAGCAATCGGGTACCAGAGAGCTGAGCTGAGAGA
AGAAACGGAAGAAAGAAAACCTGGGCAACTGGAGTGGCAGAGCTTGGGGACCTGTGAAGATCCGTAC
CTGATGTAGAGCACAGCACCCTCTCGCACACAGCGCTGCCAGCCGATGGGGACAGATGTGGCCACAG
GGCCCCCAGCTCTGTCTGCTCCACTGCTCTCATTGCCCCCATTCATTGTGTGTAATCAGCTCCCGCGTGC
CTGATAACACAGACATCCATGTAGGCACTAGGAGGATTAAACTGAGCGAAGTACCTGAGAGAGGACT
CCAGGAAGGCAAAGGTTGTGGGGAGACCCAAGAAAACTCAGGGGAAGCCCCAGAGGAGCAAGGGGA
ACCCCTCAGCTGGGAGGGAGACTGAGGAGTCCTCTGCCGTATCACCACAGCTACCTGAACATCTCTGC
AAACATTGTGGGGTCCAGTCTAAAGCCGGGGCCACCAGCTTAGCCCACACCTGCAACGCAAGTAGAG
AGATGGGACTGTCAGGAGTCTGCCCAACTCTAAAGAGCAGCACAGCCCAGCACCCTCTCAAAAGAAA
GATCCTTAACAATGTGAGGCAAGCACCTATAAAGATCTCGGGGATCTGCTGGCTTAGTCTACAACCAT
CCTTTCCAAGCCCAGTCCCTAGGGACCAGAGGGTCAGAACCTCAGCTCCCTGGGACCAAGAAAGTCTA
GCCCAATTCTTAGGCATATGAAGGGCAAGGCCTCCCTCCATTCGGAGTAGAAGACTTCAACCAGGAGT
ATGGGCACATTCCCATAATGCTAGGCCACTAGGAAGTTTCCCAGAGTGTCTGGCTCCTGTTGTTCTCTA

CCCAGGAAGATGATATAAAAAGTACAGATTGCCCCACAGGTCACCAGGCAATTGCCCAACTGCCCGCT
GGGGTGGTACCAGCAGAACTCCCCCCATGCCTGCTCCATCAGACAATCGCTGATATAGTTCATTCGGG
TCTTAAGAGGTGAGGATCACAGCTCAGTCTTCTCCCAGGATCCTGGGAAGCTCCTGATTTTCCCTCAAG
CCTACGGCAAAGGGATTCAGAACCTCCTATTCCACTGGCTTCCGATTATCTCCAAGAGAGCCTGTCCTC
CGATAGAAGGGGAAGCCCCTCCAACCTGCAGGTATCCTCCCCAGCCTCACCATTGCTCCCACCCCACA
CTGGCGGCTTCCAAACTTTCCCTCTCACAACAAGGCGCCCTGTCACCCAGACTGCTTCCCTCAGCTTGA
GGAGGAGGGGAAGGCGCACGAAGTAGGAAGGAACTTGGGGAGAGGGCGGGCGGAGGGTGGGCGAA
GCACTGAGGGGAGGGCGGTGAGGAAGGCAGAAGTCTGGCAGTGAGAGGGAGAAGCGGCGGGGGCAG
GTGAGGGCGGGGGAGTGGGGATGGGGCCGGGGAAAGGGGCCGAGAGGACGCGGAGGGGGCAGAGG
GTAGGGACGGGAGGGGGAGGGGCAGGGTGGGGGGGGCCGGGCCCCGCACTCACCGCGGCCCATGGTT
CGGCCGGCCCCGCCCTGCGCAGTCGCGGCCCAGAGGGTGAGTGGGAGGGGGTGGGAGGAGGGTCGGT
GGAGCCCGAGCCTCCGGTACGGCCCCGGCCGGTCCTAGCAGGAAGCGCCGCTGCCGCCGCCGCGGAT
CACCGAGCGGCCTCCCGCGCATGCGCCATGGGGGCCAGGGGCAGCCCTGGGGATTCCGTTCCCAGAA
GGCACTGCGCAGGCTGCTGCCGCTGCCGCCGCCGCCGTCGCTGCCGCAGCCGCCGCCGCAGGAGCCGC
CGCCTCCGCCGCTGCCCGGACGGGAGAGGGGCGGGGCCGGGCCCCCGCCCAGCGGACAGCGACGAGC
CAACTGGAGGGGCTGCAGTGCGCATGCGGGAGCGCGCCTACCCCTCCCCCACAACCCCCCATTAATCC
CCAAGAGAACAAACACCCCACGCGGCCCCCCCGCCCTCCGCGGAAGGATCTGCGCCTCTTCCCAGGCC
ACTGGTGGCCAATCCCAGCCCTCCTCAGGGAGGGGCCCCTTCCAAAGCCACAGTCTGTTGCGGGAACC
AGGAATGCCAGGTCCGGGAGGGGCCAGGCCCCGAAAGATGAAAGTCGCGACTTGCCCTGCCCCGCCC
CAAAGGCTTCCCGGGTAGTGTGCTGGGACTTGACCCGCCTCCCCAGGTCAACATGTCACAACACGACC
TCAGCCTGTCAAGTCACATGACCTCTGCCTGTCACTGACAACCTGGTCTGTCTTTAGGCCAGGGGAGAC
CATCTGGTCCACCCTCCACACTACCCCCATTTTCAGAGAGGAAATGGAGGTGTGGAGCAGCGGAGCAG
CCTAACCAAAGACCTGTCATTGCCACACCACTCCAGTCACAACCGGCCTTGTGACAGTTTCTACTTAAA
CTCTGGCTGCCCCCATGAGGGTATGACAGGATATGTAACAGGCCACCACATGGCCTCTGTTTTCAAGG
CACAAGAAAAATACTTTGCATTCTGGGTGGTGCGTTTTACTTTTGGTCAAGTTGCCTCCAAGTTCATGG
TTCTCATTTTACCTTCACAACTTTTATCTTTAATTTACAAACAAGGACGGTAAGATCCCTTGGCAAGTG
ATGGGGAGTTGAGTCTAAAATCGCGGCTCCGGTCTCCCCAGTCCAGGGCAGTCCTGCGCTGAAGTGCA
CTCACACGTCCCGGGATGAGACTCCGCTGTAAATCTCGACCCGAACACCTAGGAGGCAGGCCTGTGCC
GCGCTCCTCACACAGGGAGTCACAGGACTTGCTGTCTGGTACAGCAAGAGAGGGTCTTGGAGAAAGC
CATAGGAGTACCGTGGGGGAAAATAGGTGGCCAAACAGAATCGGGTCCACTGGGCAAAATACATGAC
TTCTGGCAGGCTGAGGGCTTACCAACTACAAGGTAAAAGTCAAGGGCGCTGTTCTGAGAGAAGCGGA
AAATGACTACCAAGGGATTCCAGTACCTCGGCCCCTTTTGGGAGATTTACGGGGCTAGAACAGGAGAC
CACCCCCGTTTTTTTGTTTGTTTGTTTTGTTTTTTGTTTTTGGTGAAACGTAGTCTCGCTCTGTCACCCAG
GCTGGAGTGCAGTGGCGCGATCTCGGTCACTGCAACATCCGCCTCCAGGGTTCAAGCGATTCTTCTGC
CTCTGCCTCCAGAGTAGCTGGGATTACAGGCGCGCATCACCACACCCGGCTAATTTTTGTATTTTTAGT
AGAGACGGGGTTTCACCATGTTGGTCAGGCTGATCTCGAACTCCTGACCTCAAGTGATCCGCTCTCCTC
AGCCTCCCAAAGTGCTGGGATTACAGGCGTAAGCCACTGAGCCCGGCCAGGAGACCTCTTTAAGAAG
ACTCGAGATGTCGACAATCCCAGTGGATGGATACCAACTTTAAAAAGAAAAGTTCAAAAGGCCTATGT
GCCCATTAGCTGGGAGGGGCCAAGAAATATGGGAGTCCCTTATAGTGGGGGTAAAACGGCGGGTAAA
GCTAGGTGGGCGGAACAGCAGCTTCTGGGGGAGACAAGCGGCAAAGAGGCTCACGACCGACTAGGGG
CGACCAAGGCTGATAGCCGTTGGGGCCGTTGGGCGCCGGGAGCTGGCGCCCCGCCCTCTCTCCTCTCC
CCCACCCTCCGCACCTCCCACCACCCTCGGTGTCCCCTGCGCGTGCGCGTGCAGACACCGGTTGCCAA
ACATTGCATCATCCCCGCCCCCCTTTCCTCCCCTCCCCCCCCGCTACACTCCCCTCCGCGGCGCGCATG
ACTCACCCACCTCCTCCGTGAAGCCTCGTGACCCAAAGCCACTTCCGGGTCCGACACTACGTCGACCC
CCTAGCGAGAGGGAGCGACGGGGGCGGTGCCGCGGGGCTCCTGAGTGGCGGATGCGAGGGACGGGG
CGGGGCCAATGCCGGCGTGCCACTTTCTGATTGGTAGGTTTTGGGGTCCCGCCCCTGAGAGGAGGGCA
AGGCCATGGTAAAAGATTACAGCCAGGCGCTCCCGAGGTC +1→ tancription initiation

CCAAT and 3´-TAACC-5´/5´-ATTGG-3´: NF-Y NF-Y serves (amongst other functions) as a co-factor for ATF6 and binds to the CCAAR part of both ERSEs.

CCAATGCCGGCGTGCCACTTTCTGATTGG: ERSE2 (5`→ 3´) und ERSE1 (3´→ 5´)

GGGGCGGGGC: SP-1, 94.5%
CCCCGCCCC: SP-1, 95.9%
GCCCCGCCC: SP-1, 94.5%
GGGGCGGGGC: SP-1, 94.5%
GGGGCAGGGT: SP-1, 95.9%

ACTTCCGGG: c-Ets-1, 94.1%

ATGACTCACCCA: AP-1, 93.4%

CCTCCGCG: MZF-1, 93.0%
CCTCCCCC: MZF-1, 93.0%
AGTGGGGA: MZF-1, 100.0% (Reverse)

ATTGCATCA: AARE1
GTTTCACCA: AARE2

TTGGGCGCC: v-Myb, 96.8%
GTTGGGGCC: v-Myb, 96.8%

GCTGATAGCC: GATA-2, 94.5% (also GATA-1, 93.1%)

CCTCCCAAAGTG: Lyf-1, 100%
TTTGGGAGA: Lyf-1, 98.7%

TCAAGTGA: Nkx-2.5, 100%

ACCACA: AML-1α, 100%

CGATTCTTCTGCC: HSF2, 93.6%

GTTTGTTTGTTT: HFH-2, 95.4%

AGTGATGGGG: GATA-1, 93.1%

GCCTAACCAAAGA: C/EBP, 96.2%

GAGACCATCTGGTCC: Tal-1a, 92.4%; Tal-1b, 92.2%

TTGCGGGAA: STATx, 92.3%
TTCCCAGAA: STATx, 96.2%

NCGTG = HRE consensus sequence, where N can be A or G, has been reported in Wikipedia and in various publications)
5´- GCGTG-3´ HRE consensus sequence
5´- ACGTG-3´ HRE consensus sequence

Fig. S3. Sequence of the 10.0-kb 5´-flanking region of the human *CHOP* gene.

High scoring (>90%) transcription factor binding sites in the 2.7-kb 5´-flanking region of the human *CHOP* gene are depicted, in addition to the known and well conserved ERSE - (ER stress-response element) and AARE (amino-acid response element) regulatory elements. NF-Y, HRE and MZF-1 regulatory elements are also found in the very distal region of the *CHOP*

promoter. Abbreviations: NF-Y: Nuclear transcription factor Y; SP-1: transcription factor SP-1; c-Ets-1: Protein c-Ets-1 (or p54); AP-1: Activator protein 1 (or c-Jun); MZF-1: Myeloid zinc finger 1; HRE: Hypoxia response element; v-Myb: Transcriptional activator v-Myb; GATA: GATA family of transcription factors (GATA1 and-2); Lyf-1: Lymphoid transcription factor Lyf-1or DNA-binding protein Ikaros; Nkx-2.5: homeobox protein Nkx-2.5; AML-1α: Acute myeloid leukemia 1 protein or Runt-related transcription factor 1; HSF2: Heat shock factor 2; HFH2: Hepatocyte nuclear factor 3 forkhead homolog 2; CEBP: CCAAT/enhancer-binding protein; Tal-1a/1b: T-cell acute lymphocytic leukemia protein 1a/b; STATx: Signal transducer and activator of transcription.

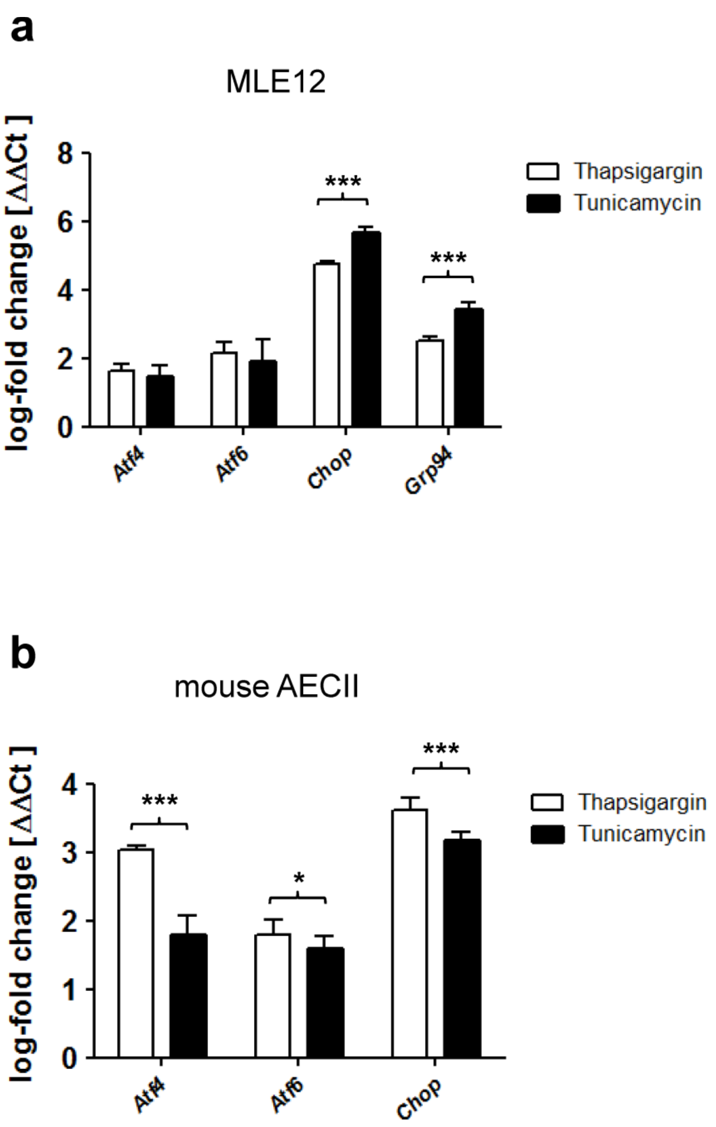

Fig. S4. Comparison of the effects of thapsigargin- and tunicamycin-treatment on ER-stress induction in MLE12 and primary mouse alveolar epithelial type-II cells (AECII).

(**a**, **b**) MLE12 cells (**a**) or primary mouse AECII (**b**) were treated for 22h with vehicle (0.025% DMSO), thapsigargin (1 µM/ml) or tunicamycin (1 µg/ml), followed by quantitative RT-PCR analysis for *Atf4*, *Atf6* and *Chop* (and *Grp94* in MLE12). *B2m* served as reference gene. From real-time PCR data, dCt values were calculated as dCt = Ct[reference]-Ct[target gene] using the Ct values of *B2m*. The $\log_2$ fold-changes are given by the ddCt values, where ddCt = dCt[treatment] - dCt[empty-vector-transfection]. All Ct values were measured in triplicate. Data are expressed as means ± SD, from n=2 independent experiments.

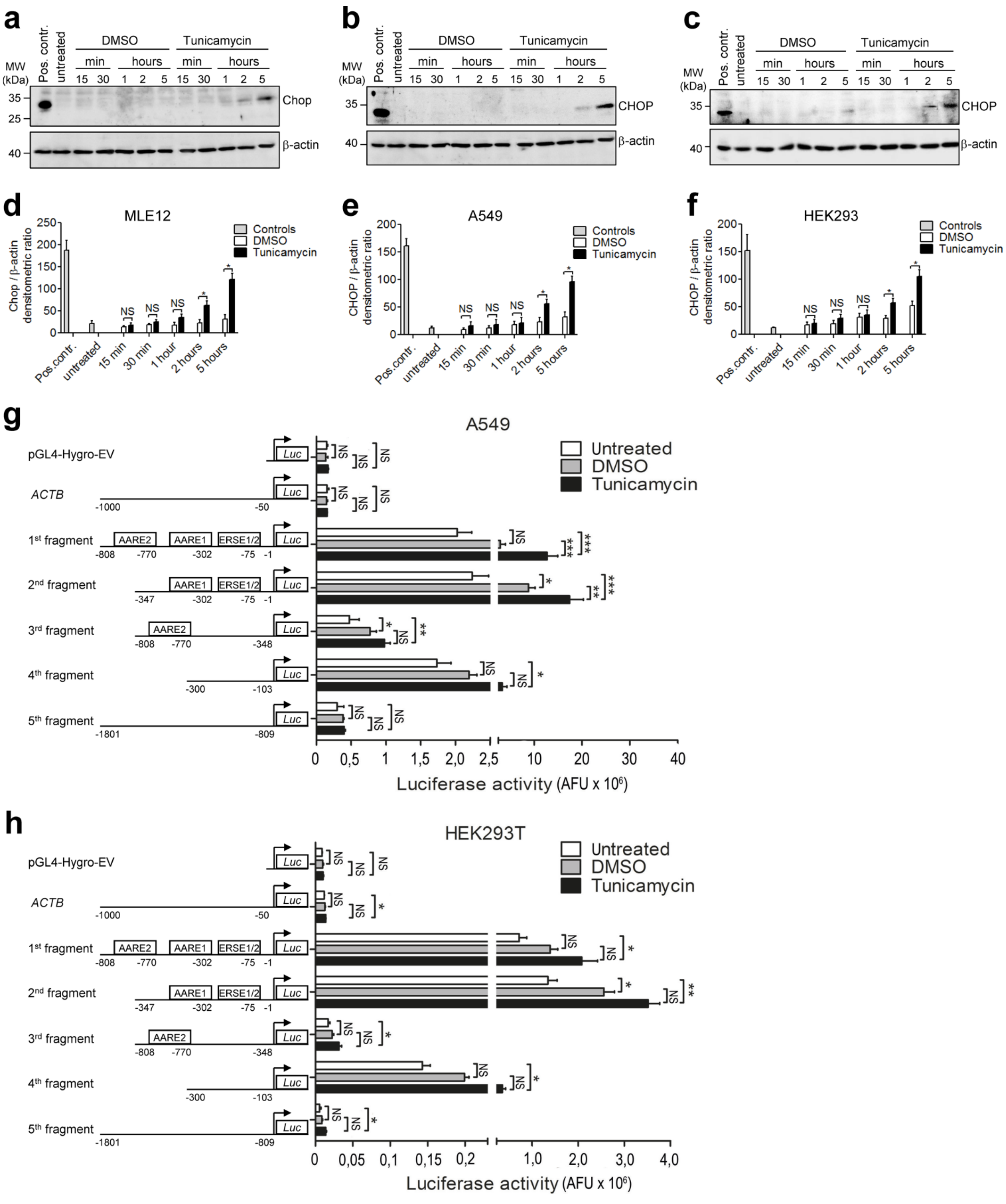

Fig. S5. Analysis of the human *CHOP* promoter in different epithelial cell lines.

(**a**-**f**) Time-dependent induction of Chop/CHOP in different epithelial cell lines MLE12 (**a**), A549 (**b**) and HEK293T (**c**) in response to 2 μg/ml of tunicamycin. β-actin served as loading

control. Each immunoblot was performed three times. The protein levels for induced Chop/CHOP in MLE12 (**d**), A549 (**e**) and HEK293 cells (**f**) were densitometrically quantified by using ImageJ software. For normalization, β-actin expression was used as a control. Data are expressed as means ± S.D., from n=3 independent experiments, with analysis by unpaired Student's t-test. *P < 0.05, NS = nonsignificant. Abbreviations: Pos. contr. = positive control, 24h-treatment of MLE12, A549 or HEK293T cells with 1 μg/ml of tunicamycin.

(**g**, **h**) *Luciferase* reporter gene-assays of cloned promoter constructs containing five different fragments of the human *CHOP* promoter or one *ACTB* promoter fragment in A549 (**g**) and HEK293T cells (**h**). All fragments including pGL4-Hygro empty-vector (EV) were transiently transfected together with a vector containing a *β-Galactosidase* reporter gene into A549 (**g**) or HEK293T cells (**h**), followed by tunicamycin (2 μg/ml) or vehicle (0.02% DMSO)-treatment 24h after transfection. After 2h, *Luciferase*- and *β-Galactosidase* assay were performed as described in the Methods. Results are presented as normalized *Luciferase* activity. Means ± S.D. are shown, from n=3 independent experiments, with analysis by Bonferroni's Multiple Comparison test. *P < 0.05, **P < 0.01, ***P<0.001, NS = nonsignificant.

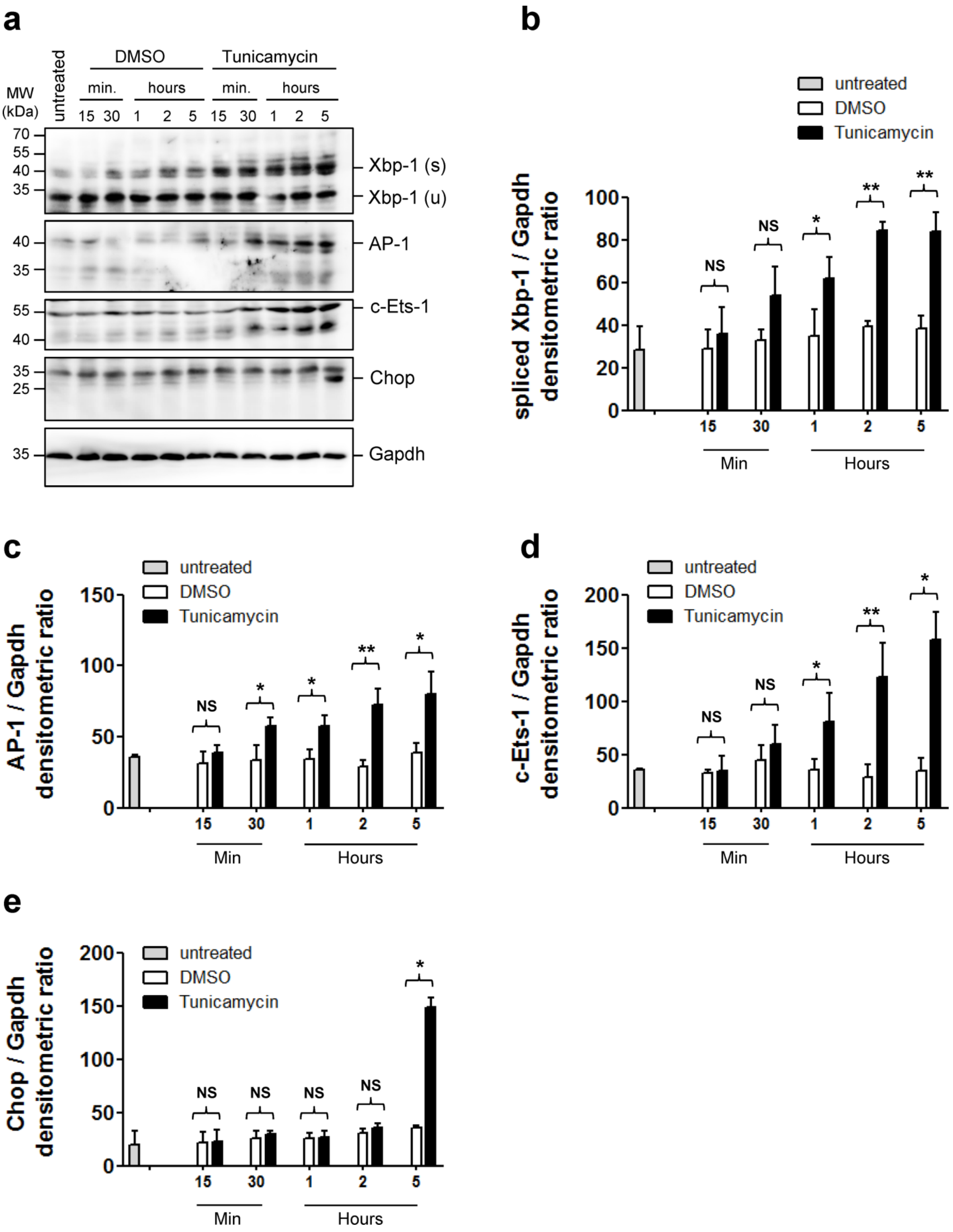

Fig. S6. AP-1 and c-Ets-1 are upregulated under ER-stress conditions in primary mouse alveolar epithelial type-II cells (AECII).

(**a**-**e**) Primary mouse AECII were treated with 0.02% DMSO (vehicle) or 2 µg/ml tunicamycin for the indicated time periods. Protein lysates were subjected to western blotting for indicated antibodies (**a**). The protein levels for spliced Xbp-1 (**b**), AP-1 (**c**), c-Ets-1 (**d**), and Chop (**e**) were densitometrically quantified by using ImageJ software. For normalization, Gapdh expression was used as a control. All data are expressed as means ± S.D., from n=3 independent experiments, with analysis by unpaired Student's t-test. *P < 0.05, **P < 0.01, NS = nonsignificant.

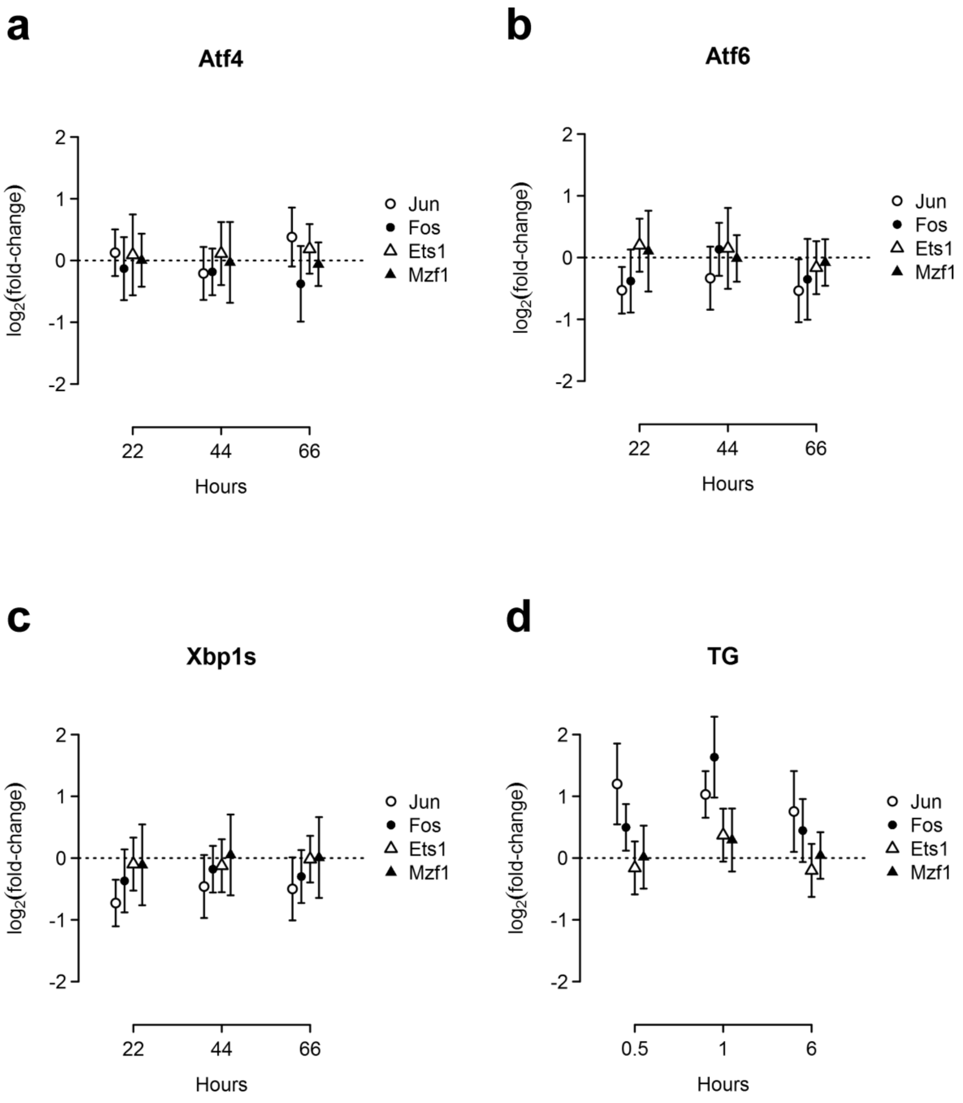

Fig. S7. Microarray analysis showing the differential expression of *Jun*, *Fos*, *Ets1* and *Mzf1* using RNA from MLE12 cells after overexpression of the UPR transcription factors Atf4 (**a**), p50Atf6 (**b**) or spliced Xbp1 (Xbp1s) (**c**), against empty-vector transfection, in comparison to thapsigargin (TG, 1 μM/ml)-treated cells (**d**) (n=6 experiments per condition for time periods 22h, 44h, 66h; and n=4 for the 0.5h, 1h and 6h treatments with TG, and n=12 for the respective control). Error bars are 95% confidence intervals. The microarray as described in Fig. 1a and Fig. 1b (main manuscript) and Fig. S1 was used for expression analysis of indicated genes. Because c-Fos may form a heterodimer-complex with the *Jun*/AP-1 transcription factor at various gene promoters, its gene expression under conditions of ER-stress was of interest.

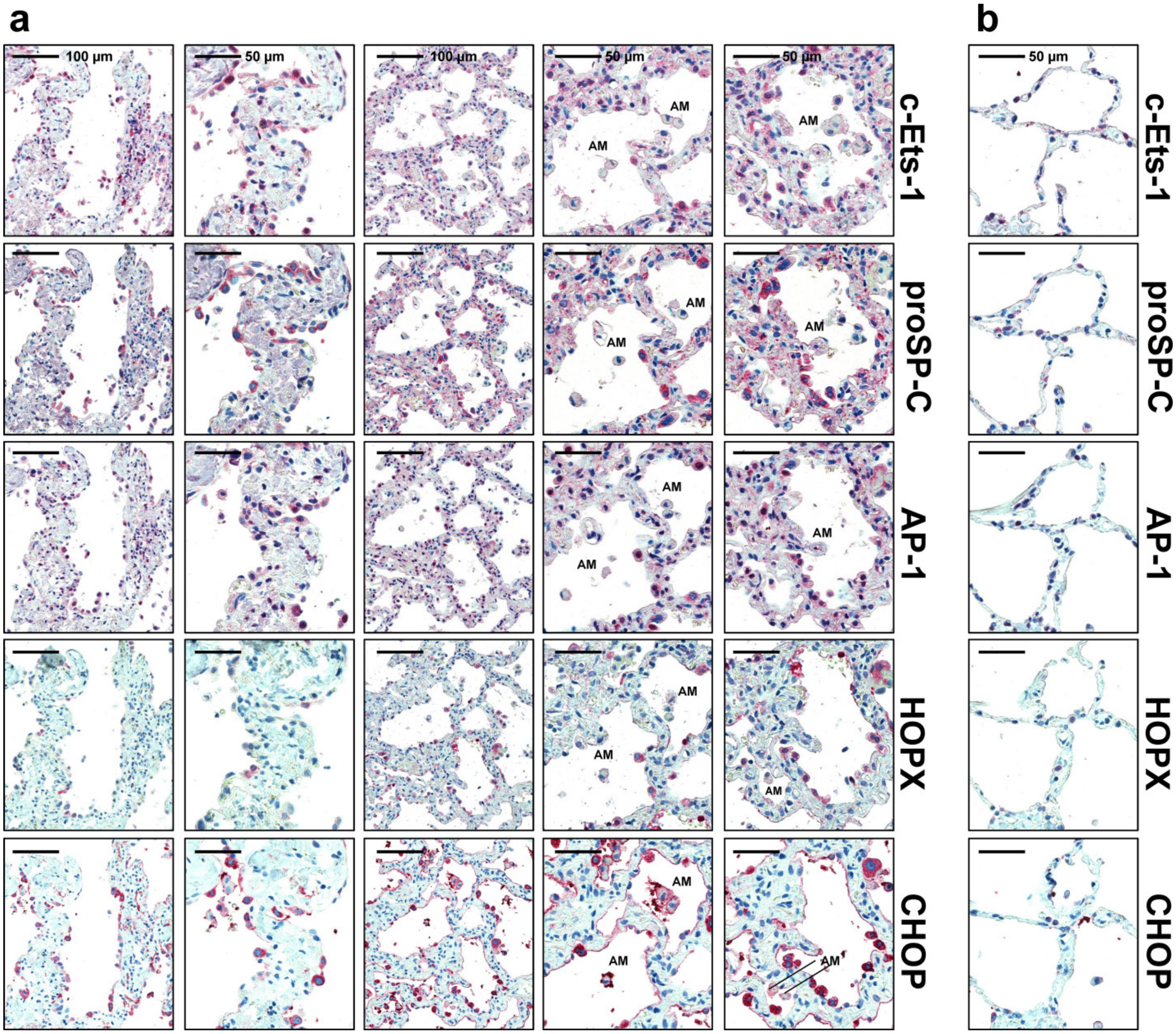

Fig. S8. AP-1, c-Ets-1 and CHOP are induced in AECII from patients with IPF. Immunohistochemical staining of serial sections of IPF- (**a**) and normal donor lung tissues (**b**) for c-Ets-1, proSP-C (AECII-marker), AP-1, HOPX (AECI-and AECII marker) and CHOP. (**a**) In IPF, proSP-C expressing AECII indicated robust overexpression of c-Ets-1 and AP-1 in the nucleus and cytoplasm, and co-localized with induced CHOP expression. Alveolar macrophages (AM) of IPF-lungs indicated only faint expression of c-Ets-1 and AP-1, and no significant expression of CHOP. It should be noted, that the rabbit polyclonal anti-CHOP antibody (#5554 from CST) sometimes appeared to stain (unspecifically) mucuos debris in the alveolar spaces.

(**b**) In normal donor lungs, AP-1 and c-Ets-1 were expressed at low basal level in AEC, and occasionally found at low-to-moderate level in AM. Minimal or no immunostaining for CHOP was observed in any cells of donor lungs. Results are representative for n=6 IPF-patients and n=3 organ donors.

a

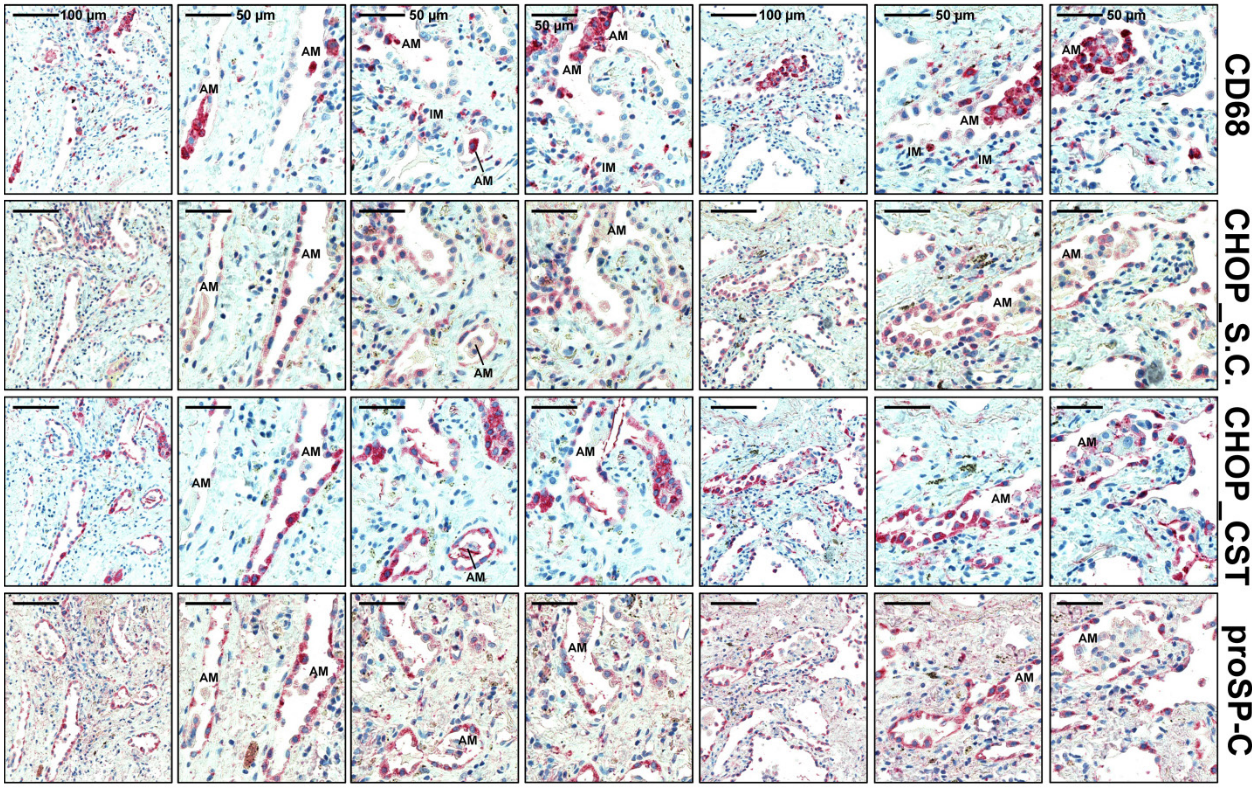

b

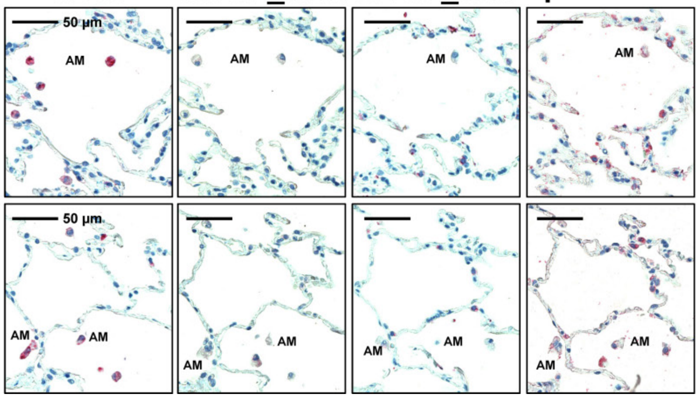

Fig. S9. CHOP is exclusively induced in AECII, but not in AM from patients with IPF.

Immunohistochemical staining of serial sections of IPF- (**a**) and normal donor lung tissues (**b**) for CD68 (macrophage marker), CHOP (mouse monoclonal anti-CHOP antibody sc-7351 from Santa Cruz, as well as rabbit polyclonal anti-CHOP antibody #5554 from CST) and proSP-C (AECII-marker). (**a**) In IPF, both different anti-CHOP antibodies indicated predominantly robust immunoreactivity in proSP-C expressing AECII, but not in CD68 expressing alveolar macrophages (AM). (**b**) No or only minimal immunostaining for CHOP was observed in any cells of normal donor lungs. Results are representative for n=6 IPF-patients and n=3 organ donors.

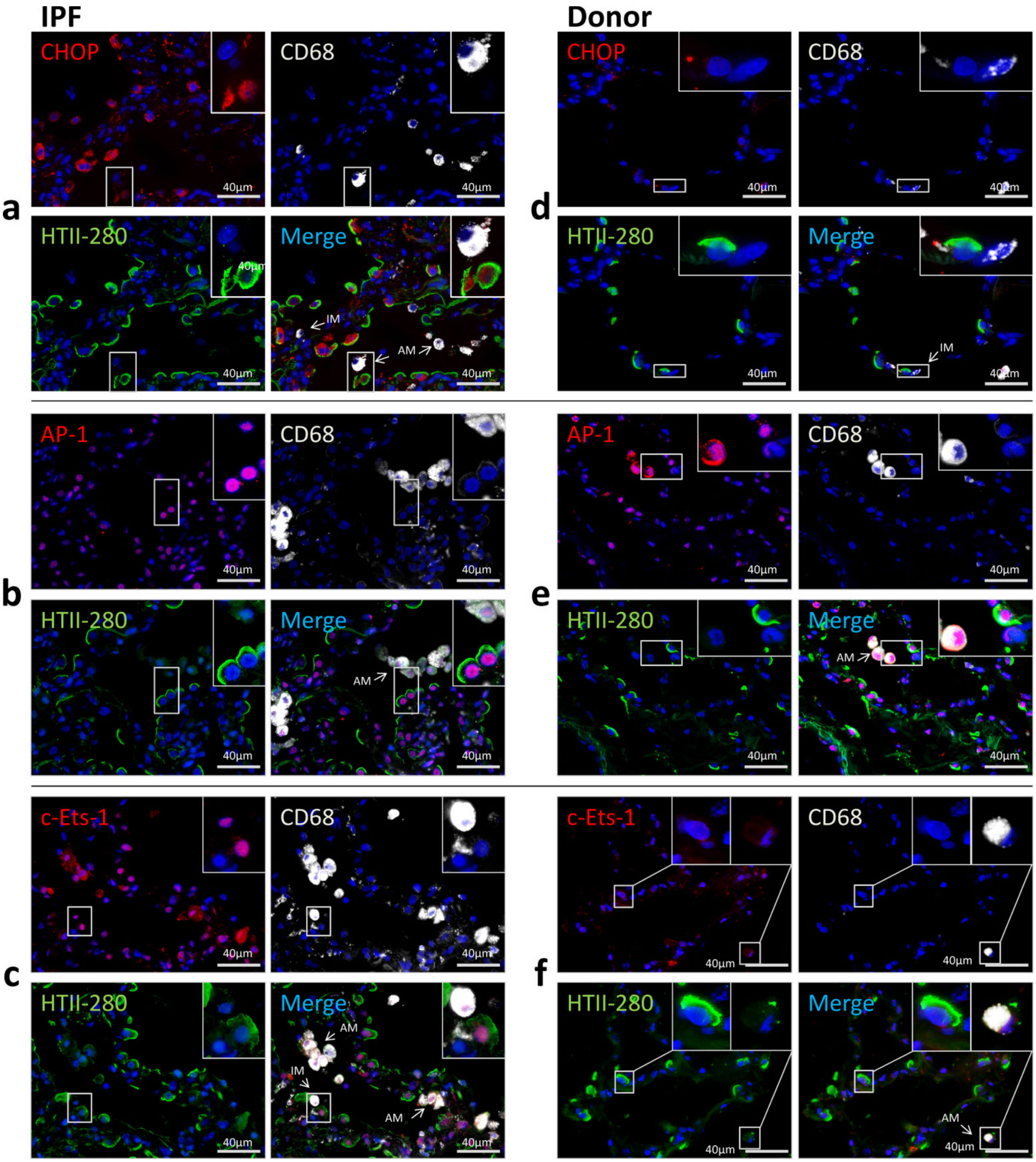

Fig. S10. CHOP, AP-1 and c-Ets-1 are exclusively induced in AECII, but not in AM from patients with IPF.

(**a-f**) Immunofluorescence staining for CHOP, AP-1, c-Ets-1, CD68 (macrophage marker) and HTII-280 (AECII marker) of lung tissue sections from patients with IPF (**a**, CHOP; **b**, AP-1 and **c**, c-Ets-1) and organ donors (**d**, CHOP; **e**, AP-1 and **f**, c-Ets-1). Nuclei were counterstained with DAPI (blue color). In IPF, HTII-280 expressing AECII (green color) indicated nuclear overexpression of CHOP (**a**), AP-1 (**b**) and c-Ets-1 (**c**) (red color). In contrast, CD68 positive

interstitial [IM] and alveolar macrophages [AM] (indicated in white color) of IPF lungs indicated no significant expression of CHOP (**a**), AP-1 (**b**) or c-Ets-1 (**c**). In normal donor lungs, CHOP (**d**) and c-Ets-1 (**f**) were absent in HTII-280 expressing AECII and CD68-positive macrophages. Interestingly, nuclear expression of AP-1 was occasionally observed in HTII-280 expressing AECII and CD68-positive macrophages of donor lungs (**e**), presumably due to its multifunctional role and involvement in various biological pathways, including (non-pathogenic) homeostasis-related signalling pathways. Results are representative for n=3 IPF-patients and n=3 organ donors.

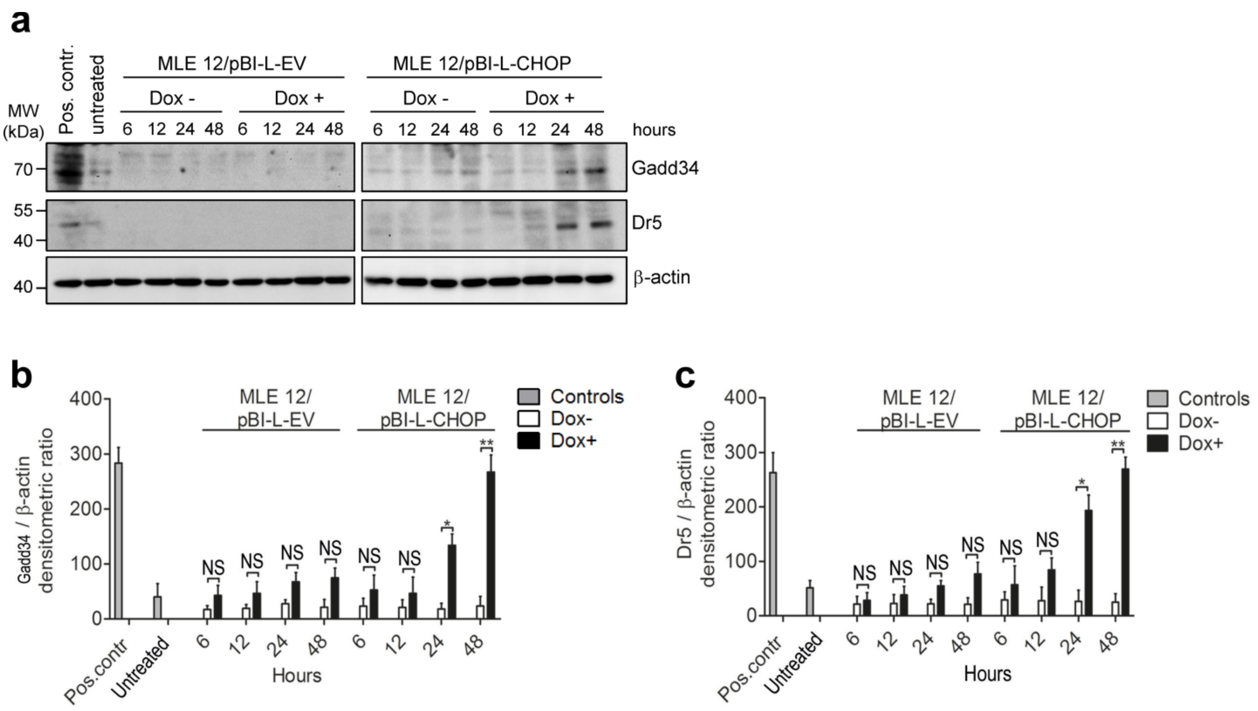

Fig. S11. Chop-overexpression induces expression of pro-apoptotic target genes in lung epithelial cells *in vitro*. (**a**-**c**) Stably-transfected MLE12/pBI-L-EV (empty-vector-control) and MLE12/pBI-L-CHOP cells were treated with 1 µg/ml doxycycline (Dox+, for transgene induction) or left untreated (Dox-, control), followed by quantitative immunoblot analysis for Gadd34 and Dr5. β-actin served as loading control. (**b**, **c**) Densitometric quantification for Gadd34 (**b**) and Dr5 (**c**), respectively. Means ± S.D. are shown, from n=3 experiments, with analysis by unpaired Student's t-test. *P < 0.05, **P < 0.01, NS = nonsignificant. Abbreviations: Pos. contr. = positive control, 24h-treatment of MLE12 cells with 1 µg/ml of tunicamycin.

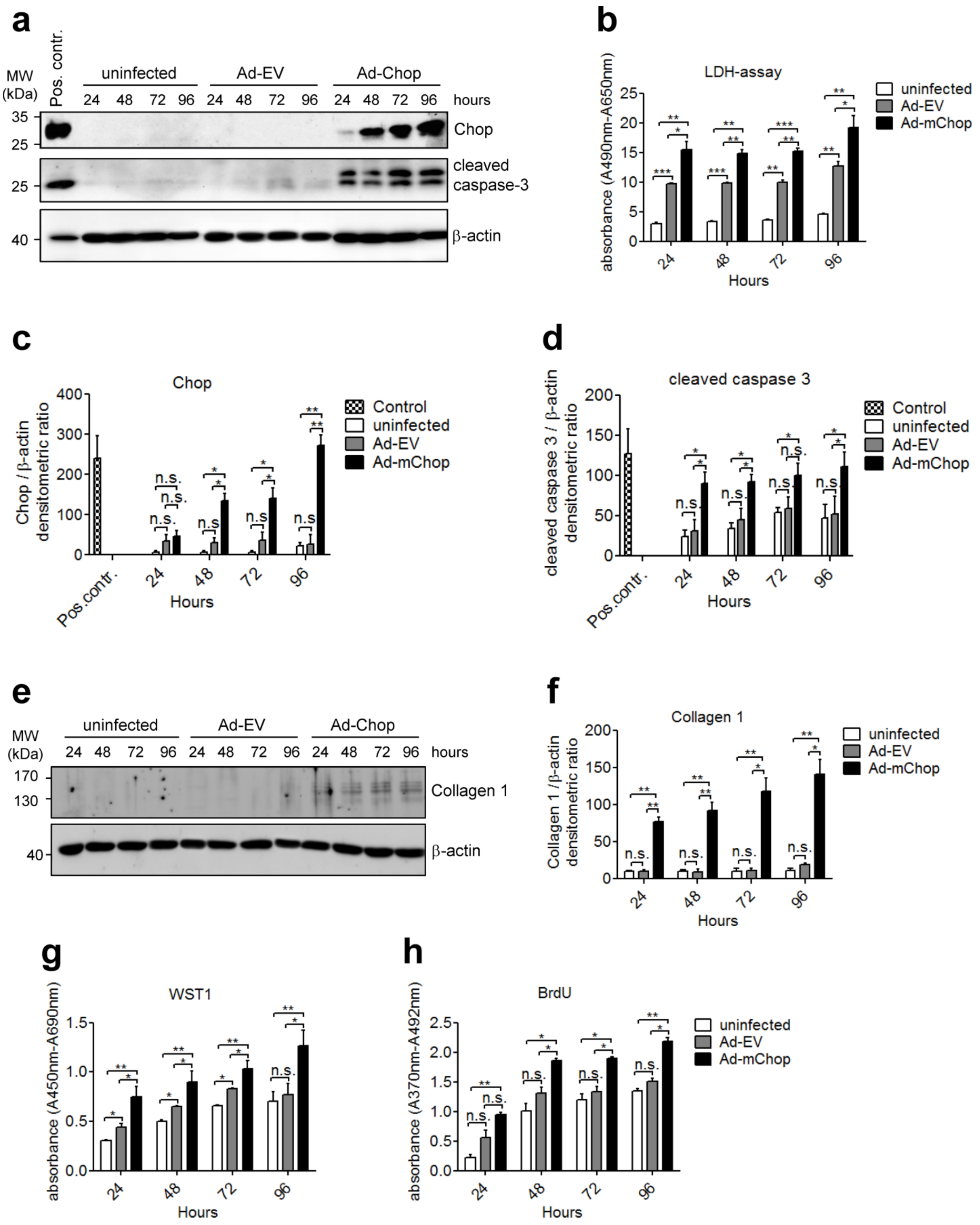

Fig. S12. Effect of Chop-overexpression on apoptosis of AECII and fibroblast proliferation *in vitro*. (**a**) Primary murine AECIIs were infected with adenoviral vectors Ad-EV (empty-vector-control), Ad-Chop or left uninfected, followed by quantitative immunoblot analysis for

Chop and cleaved caspase-3. β-actin served as loading control. (**b**) LDH assay quantifying AECII cell death in response to Chop-overexpression. (**c**, **d**) Densitometric quantification for Chop-expression (**c**) and caspase-3-activation (**d**). Pos. contr. = positive control, AECII cells treated either with tunicamycin (1 μg/ml for 24h) or staurosporine (1 μM/ml for 8h), for Chop- or cleaved caspase-3-blot, respectively. (**e**) Conditioned medium from AECII cells infected with Ad-EV or Ad-Chop was applied to Mlg lung fibroblasts, followed by assessment of their collagen-synthesis by quantitative immunoblotting using anti-collagen-1 antibody and β-actin as loading control. (**f**) Densitometric quantification for collagen-1-expression. (**g**, **h**) Proliferation of Mlg lung fibroblasts by WST-1 assay (**g**) or BrdU incorporation (**h**) in response to culture with supernatants of AECII infected with Ad-EV or Ad-Chop. In (**b**-**d**) and (**f**-**h**), means ± S.D. are shown, from n=3 experiments, with analysis by unpaired Student's t-test. *P < 0.05, **P < 0.01, ***P < 0.001, n.s. = nonsignificant.
